# Rapid molecular genetic diagnosis of hypertrophic cardiomyopathy by semiconductor sequencing

**DOI:** 10.1186/1479-5876-12-173

**Published:** 2014-06-17

**Authors:** Zongzhe Li, Jin Huang, Jinzhao Zhao, Chen Chen, Hong Wang, Hu Ding, Dao Wu Wang, Dao Wen Wang

**Affiliations:** 1 Departments of Internal Medicine and Gene Therapy Center, Tongji Hospital, Tongji Medical College, Huazhong University of Science and Technology, 1095# Jiefang Ave, Wuhan 430030, China; 2 Division of Cardiology, the First Affiliated Hospital, Nanjing Medical University, Nanjing, China

**Keywords:** Genetic diagnosis, Hypertrophic cardiomyopathy, Next-generation sequencing, Semiconductor sequencing

## Abstract

**Background:**

Rapidly determining the complex genetic basis of Hypertrophic cardiomyopathy (HCM) is vital to better understanding and optimally managing this common polygenetic cardiovascular disease.

**Methods:**

A rapid custom Ion-amplicon-resequencing assay, covering 30 commonly affected genes of HCM, was developed and validated in 120 unrelated patients with HCM to facilitate genetic diagnosis of this disease. With this HCM-specific panel and only 20 ng of input genomic DNA, physicians can, for the first time, go from blood samples to variants within a single day.

**Results:**

On average, this approach gained 595628 mapped reads per sample, 95.51% reads on target (64.06 kb), 490-fold base coverage depth and 93.24% uniformity of base coverage in CDS regions of the 30 HCM genes. After validation, we detected underlying pathogenic variants in 87% (104 of 120) samples. Tested seven randomly selected HCM genes in eight samples by Sanger sequencing, the sensitivity and false-positive-rate of this HCM panel was 100% and 5%, respectively.

**Conclusions:**

This Ion amplicon HCM resequencing assay provides a currently most rapid, comprehensive, cost-effective and reliable measure for genetic diagnosis of HCM in routinely obtained samples.

## Background

Hypertrophic cardiomyopathy (HCM) is regarded as a most common inherited cardiac disorder (1/500) and the leading cause of sudden cardiac death in adolescents 0020 [1-4]. So far, over 1000 mutations in at least 30 genes have been reported to responsible for HCM, which implied an highly genetic heterogeneity and hence resulting various clinical phenotypes, ranging from asymptomatic forms to sudden cardiac death in the young [3,5-12]. Although disease-causing mutations in *MYH7, MYBPC3* and *TNNT2* had been considered to explain about half of HCM patients [8,9], the frequency of each causal variant is relatively low and most rare mutations are unique in specific families [13]. Moreover, about 10% HCM patients harbored more than one mutation and thus suffering from an earlier onset or worse prognosis [2,7]. Therefore, systemic genetic diagnosis for HCM patients was necessary and recommended by current clinical guidelines [14-17]. For instance, the identification of sudden-death-high-risk patients could benefit from an implantable cardioverter-defibrillator in primary prevention [18].

However, conventional Sanger sequencing was too laborious and expensive to content regular clinical practice [19]. Advancing high throughput next-generation sequencing (NGS) technologies have the potential to solve the problem by rapidly dissecting large regions at low cost [1,20-22]. Nevertheless, the current NGS platforms have several weaknesses, including sample scalability, sequencing time and cost of entry, which need to be addressed if these technologies are going to service clinical routine genetic diagnosis [23]. With lowest-price, shortest running time, minimum start DNA amount and flexible sequencing-chip reagents, the recent flourishing semiconductor sequencing technique is notable [21,24].

Our study provides, to our knowledge, a currently most rapid, comprehensive, cost-efficient and reliable assay for genetic diagnosis of hypertrophic cardiomyopathy in everyday clinical practice. Implementation of this method will change diagnosis and understanding of the molecular etiologies of HCM.

## Materials and methods

### HCM resequencing panel design

For the HCM resequencing panel targeted genes selection, recent ten years’ literatures, including prior genetic detection technique articles, reviews and case-reports of HCM, were carefully accessed. To recruit a maximum coverage of the mutation spectrum of this polygenetic disorder, we designed a currently most comprehensive HCM-specific resequencing panel including 30 causal genes that most frequently affected in patients with HCM (Table 1). Then, primers of overlapping amplicons covering the CDS-region and flanking sequences of each targeted gene were automated designed by Ion AmpliSeq™ Ready-to-Use custom designer platform following guide of the website ( https://www.ampliseq.com/protected/dashboard.action) (Primers for Semiconductor sequencing are presented in Additional file 1: Table S1). With the ability to perform ultrahigh-multiplex PCR reaction in one tube parallelly, the primers were mixed and provided (Life Technologies, Carlsbad, California, USA) in two primer-pools. Eventually, 97.96% of the targeted region (64.06 kb) was overlapped by 690 about 200 bp-length amplicons.

**Table 1:** Selected hypertrophic cardiomyopathy genes

**Nr.**	**Gene**	**Ensembl number**	**Chromosome**	**CDS, n**	**Amplicons, n**	**Target, bp**	**Missed**^**#**^**, bp**	Coverage^*^,%
1	MYBPC3	ENSG00000134571	chr11	32	46	3826	9	99.8
2	MYH7	ENSG00000092054	chr14	38	54	5808	127	97.8
3	TNNT2	ENSG00000118194	chr1	21	17	1286	0	100
4	ACTC1	ENSG00000159251	chr15	6	10	1134	0	100
5	TNNI3	ENSG00000129991	chr19	7	8	632	0	100
6	TPM1	ENSG00000140416	chr15	16	19	1429	0	100
7	MYL2	ENSG00000111245	chr12	7	7	501	0	100
8	MYL3	ENSG00000160808	chr3	6	8	588	71	87.9
9	TCAP	ENSG00000173991	chr17	2	4	504	0	100
10	PRKAG2	ENSG00000106617	chr7	18	22	1795	74	95.9
11	TNNC1	ENSG00000114854	chr3	6	7	486	10	97.9
12	CSRP3	ENSG00000129170	chr11	5	6	585	0	100
13	MYH6	ENSG00000197616	chr14	37	55	5820	216	96.3
14	PLN	ENSG00000198523	chr6	1	10	1503	364	75.8
15	MYOZ2	ENSG00000172399	chr4	5	10	795	0	100
16	ACTN2	ENSG00000077522	chr1	21	27	2685	0	100
17	JPH2	ENSG00000149596	chr20	6	17	2102	155	92.6
18	LAMP2	ENSG00000005893	chrX	11	19	1516	0	100
19	CAV3	ENSG00000182533	chr3	2	4	456	0	100
20	VCL	ENSG00000035403	chr10	22	37	3405	39	98.9
21	ANKRD1	ENSG00000148677	chr10	9	10	960	0	100
22	GLA	ENSG00000102393	chrX	7	13	1290	0	100
23	LDB3	ENSG00000122367	chr10	16	26	2508	0	100
24	CALR3	ENSG00000269058	chr19	9	13	1155	22	98.1
25	MYLK2	ENSG00000101306	chr20	12	22	1791	0	100
26	RYR2	ENSG00000198626	chr1	105	165	14904	74	99.5
27	CASQ2	ENSG00000118729	chr1	11	14	1200	0	100
28	FXN	ENSG00000165060	chr9	8	10	874	32	96.3
29	LMO4	ENSG00000143013	chr1	4	6	498	0	100
30	NEXN	ENSG00000162614	chr1	12	25	2028	111	94.5

Ensembl: June 2013 (GRCh37/hg19). CDS, coding sequence; *indicates in silico coverage of target sequences by multiplex PCR; ^#^indicates in silico missed bases by multiplex PCR.

### Patients and DNA sample preparation

With approval from the local ethics committee, 120 unrelated Chinese Han HCM patients confirmed by echocardiography during 2008 to 2013 with written informed consent were included in this study. Two of the 120 patients were from independent HCM pedigrees, carrying known pathogenic mutations rs121913641 and rs121913637 in the same loci (p.R719Q, p.R719W) in gene *MYH7*, respectively. Genomic DNA (gDNA) of each patient was extracted and RNase managed from peripheral leukocytes, using a DB-S kit (FUJIFILM Corporation, Tokyo, Japan) according to the manufacturer’s instructions. The purified gDNA was then checked with electrophoresis to avoid fragmental degradation and RNA pollution.

### Library preparation and sequencing

**Figure 1:**
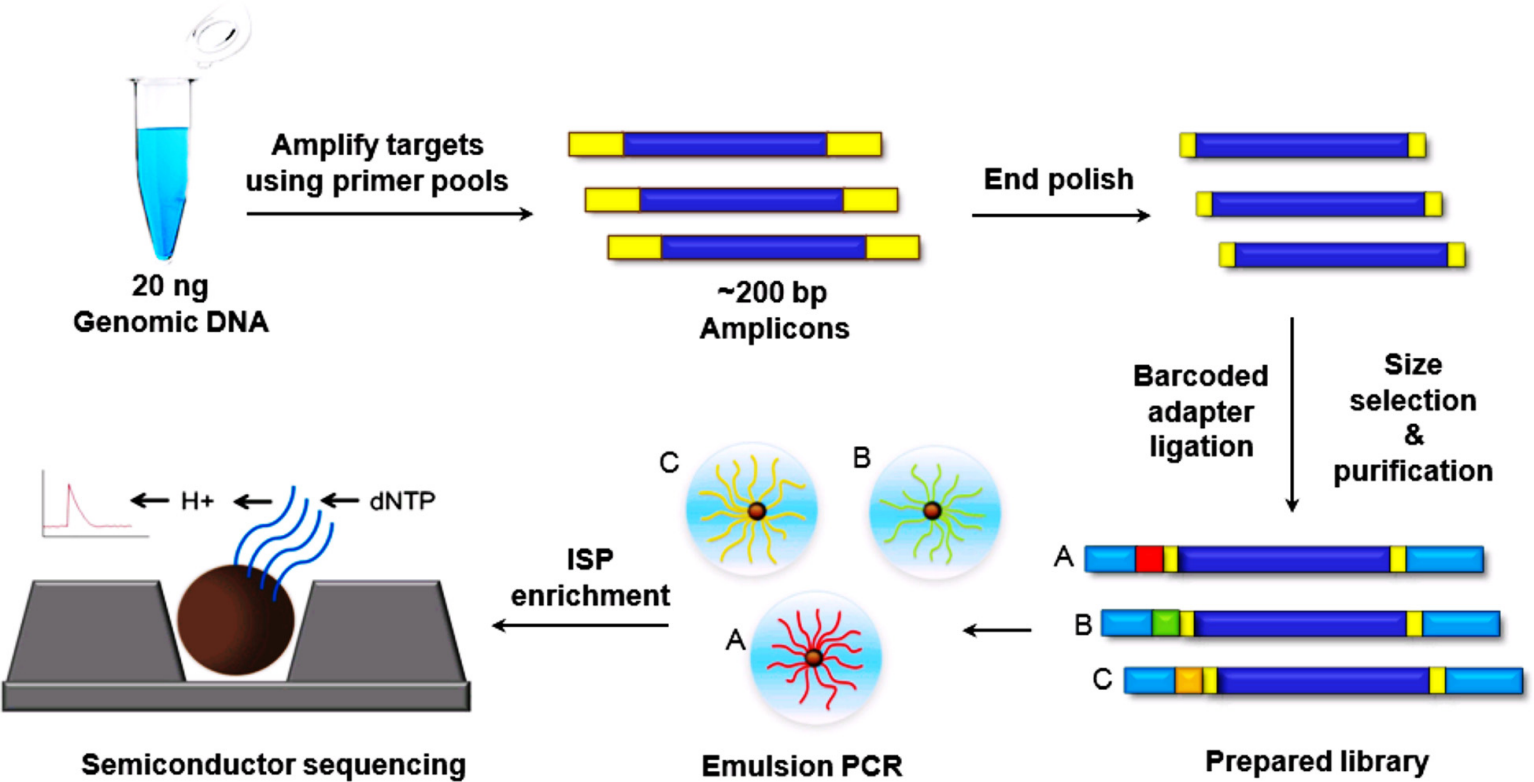
**Semiconductor sequencing flow diagram.** Amplicons of 30 HCM genes are amplified by using 20 ng input genomic DNA and two primer pools. The about 200 bp amplicons are end-polished, barcodes and adapters ligated, purified to become prepared libraries. Emulsion PCR is conducted by Ion One Touch to generate ISPs for semiconductor sequencing. ISP indicates Ion Sphere Particles.

Ion Torrent adapter-ligated libraries were builded using Ion Ampliseq™ Library Kit 2.0 (Life Technologies) following the manufacturer’s protocol within about 5 hours. Briefly, 20 ng gDNA for every sample was quantitated by Qubit 2.0 fluorometer (Invitrogen, Carlsbad, CA, USA) for multiplex PCR amplification with each of the two primer-pools, respectively. The resulting amplicons of the two primer-pools were mixed together, and then ligated to barcodes and Ion Torrent adapters (Life Technologies). Subsequently libraries were purified with AMPure XP beads (Beckman Coulter, Brea, CA, USA) using 5-cycles of PCR amplification and further purification, followed by quantification by Qubit 2.0 fluorometer. In order to increase efficiency and reduce costs, sixteen uniquely barcoded libraries were combined together with equal molar ratios for one 318 chip. Subsequent emulsion PCR and enrichment of the sequencing beads of the pooled libraries was performed using the OneTouch system (Life Technologies) according to the manufacturer’s protocol within about 5 hours. Finally, 500 Flows (125 cycles) sequencing was done on the 318- chip using Ion PGM 200 Sequencing Kit (Life Technologies) on the Ion Torrent Personal Genome Machine (PGM) (Life Technologies) (Figure 1).

### Bioinformatic analysis

Raw data from 4.5 hours’ PGM runs were initially processed using the Ion Torrent platform-specific software Torrent Suite v3.6.2 to generate sequence reads, trim adapter sequences, align to the hg19 human reference genome, analyze coverage and call variants (Variants Caller parameter settings see Additional file 1: Table S2). Then, all variants were annotated with an online-software Variant Effect Predictor ( http://asia.ensembl.org/info/docs/variation/vep/index.html). To predict possible impact of detected non-synonymous variants in exons, all missense substitutions were scored and in-silico-function-predicted by SIFT ( http://sift.jcvi.org/www/SIFT_BLink_submit.html) and PolyPhen-2 ( http://genetics.bwh.harvard.edu/pph2/). The putative novel pathogenic variants were further confirmed that were reported neither in the 1000-Genome project database nor in the NCBI dbSNP database. To calculate the importance of novel mutations, conservation test was performed in patients and thirteen other species around the mutated position by COBALT algorithm ( http://www.ncbi.nlm.nih.gov/tools/cobalt/cobalt.cgi).

### Criteria for functional mutations

To determine a functional variant, the variant should be a Sanger sequencing validated coding variant. If the variant is a previously reported variant, it should be causal mutations in NCBI ClinVar database or HGMD database. If the variant is a novel variant, it should in accordance with none of the following database: the 1000-Genome project database, the NCBI dbSNP database, UCSC common SNP database and the 5000 Exmoes database in Exome Sequencing Project (ESP). If the variant is a missense substitution, it should be evolutionarily conserved and predicted functional damaged by either SIFT or Polyphen-2.

### Sanger sequencing validation

All potential functional variants were validated with Sanger sequencing in an Applied Biosystems 3130 capillary sequencer using individual primers in both directions to obsolete false positive errors. The technically uncovered 1304 bp regions of the 13 targeted genes were carefully sequenced directly by Sanger sequencing. No more functional variant was detected in all 120 patients. To evaluate the sensitivity and false-positive-rate of this panel, we randomly selected seven genes (*MYH7, MYBPC3, ACTC1, PRKAG2, MYOZ2, ACTN2, JPH2*) and sequenced all exons and flanking regulation regions directly by Sanger sequencing in eight subjects from the 120 HCM patients.

## Results

### Study population

One-hundred-and-twenty unrelated patients with HCM were studied. The mean age of male (85/120) was 45 ± 16 years (1, 78) and the maximal wall thickness was 18.34 ± 5.1 mm (14, 46). For female (35/120) the mean age was 51 ± 17 years (15, 86) and the maximal wall thickness was 19.73 ± 4.8 mm (13, 41). The mean left ventricular ejection fractions of male and female are 58 ± 14 and 62 ± 13, respectively. In the 120 HCM patients, 24.2% (29/120) are hypertrophic obstructive cardiomyopathy and 19.2% (23/120) have positive family history. (The detailed characteristics of HCM Patients were shown in Additional file 1: Table S3).

### Sequencing output and coverage

**Figure 2:**
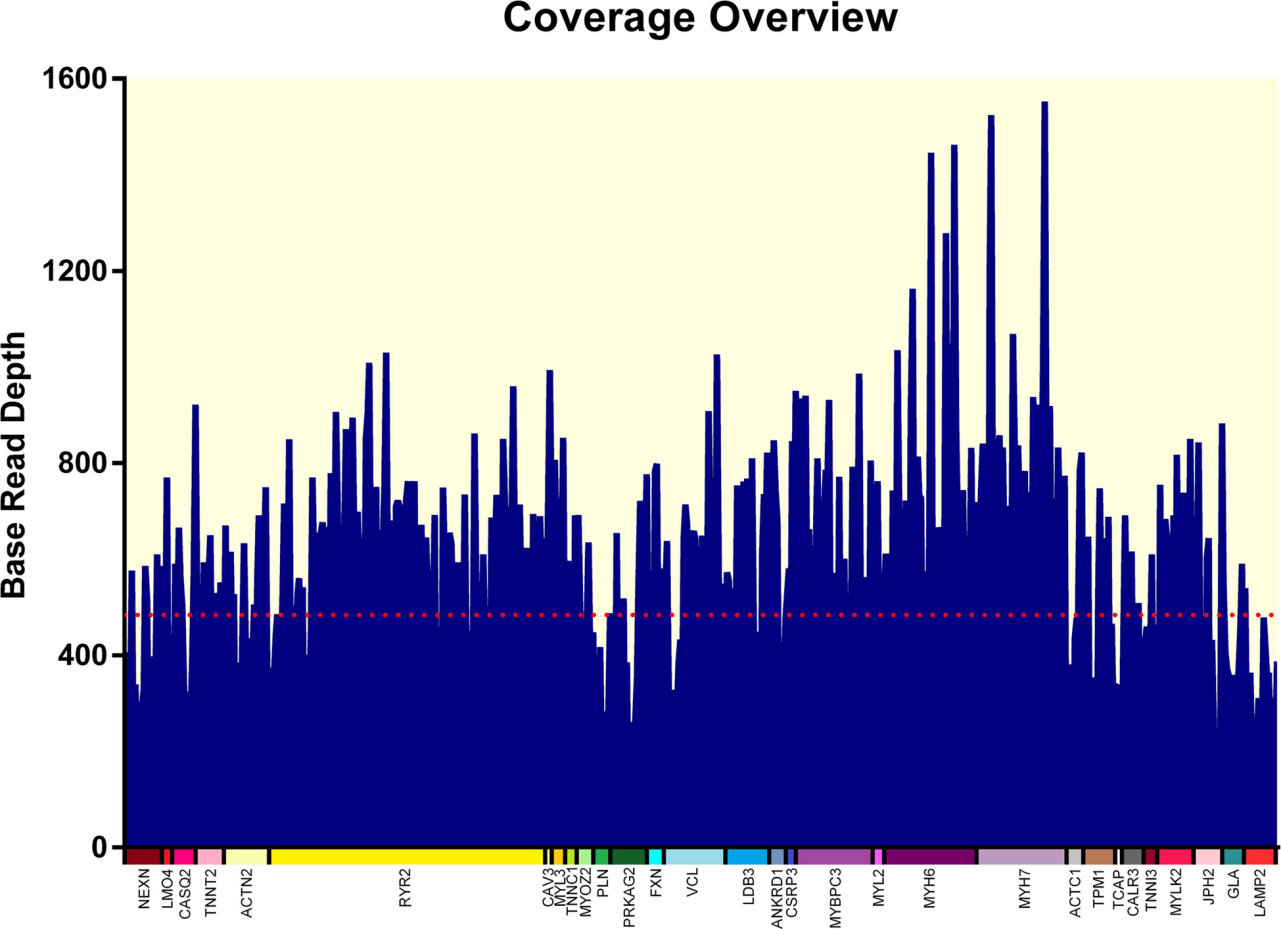
**Sequencing coverage overview of the 30 HCM genes for 120 samples.** Blue graphs represent the distribution of coverage of the 30 HCM genes for 120 samples. The dashed line indicates the mean coverage (490X) over the enriched 30 HCM genes (color blocks).

The sequencing of selected regions of 30 HCM-associated genes on the Ion torrent PGM achieved an average output of 595628 mapped reads and 95.51% on target per sample in the 120 HCM specimens. In summary, 99.55% of all target amplicons was covered at least once, 96.98% amplicons was covered at least 20 times, 91.95% amplicons was covered at least 100 times. The mean uniformity of base coverage is 93.24% in this panel. The average read depth in the 64.06 K target region across the 120 samples was ~490 folds (Figure 2). Moreover, chip-loading-rate was improved shortly and polyclonal-rate was reduced significantly after few trails in the beginning of experiments, which result in an increase in mean coverage.

### Mutation detection and sanger sequencing validation

The Ion Torrent platform-specific software Torrent Suite v3.6.2 and online software Variant Effect Predictor were employed to align the reads sequences to the human reference genome build hg19, call variants and bioinformatical annotate. Criteria for variant identification were a read coverage of higher than 30-fold. All together, in the 120 patients, 458 known or novel variants were detected by Semiconductor sequencing and on average 80 variants per sample. After Sanger sequencing validation, except 25 variants, 433 variants were determined truly exist. Most of the 25 false-positive miscalls are insertions or deletions and detected in more than one sample. Of these 433 variants, 345 (80%) are predicted to be noncoding or synonymous, whereas 88 (20%) are non-synonymous, including missense mutation and small insertion/deletion, resulting in the change of amino acids (Table 2). Notably, we identified at least one functional variant in 104/120 (87%) HCM patients and found more than one functional variants in 12/120 (10%) HCM patients.Furthermore, the two known positive pathogenic mutations (rs121913641 and rs121913637) in the two probands were successfully identified.

**Table 2:** Sequencing summary

**Measure**	**Value**
**No. of genes**	**30**
**No. of exons**	**462**
**No. of amplicons**	**690**
**Total no. of variants**	**433**
**Deletions**	**11**
**Insertions**	**7**
**SNVs**	**415**
**Noncoding**	**240**
Intron variant	212
Splice region variant	19
3′ UTR variant	5
5′ UTR variant	4
**Synonymous**	**105**
**Nonsynonymous**	**88**
Missense variant	79
Stop gain variant	6
Frameshift variant	3
**No. of known dbSNPs**	**293**
**No. of novel variants**	**140**

SNVs, single-nucleotide variants.

### Sensitivity and false-positive-rate evaluation

To further assess the sensitivity and specificity of this HCM panel, direct Sanger sequencing of seven randomly selected genes was performed in eight selected subjects. Finally, 38 variants were detected by semiconductor sequencing, including 35 known variants and 3 novel variants (Table 3). Compared with Sanger sequencing results, 2 variants were failed to be validated. Therefore, the sensitivity of this HCM panel was evaluated as 100% and the false-positive-rate was evaluated as 5%.

**Table 3:** Variants detected by semiconductor sequencing of the seven selected genes in eight samples

**Gene**	**Position**	**Type**	**Zygosity**	**Nucleotide substitution**	**Variant frequency**	***P*-value**^**#**^	**Coverage**	**Consequence**	**Novelty**	**No. of patients**	**Validated by Sanger sequencing**
***ACTC1***	15:35084215	SNP	Het	C/G	47.94	6.3E-06	413	intron	rs3729755	5	Yes
***ACTN2***	1:236899042	SNP	Het	G/A	44.09	2.5E-05	254	intron	rs2288600	2	Yes
	1:236902865	SNP	Het	A/C	55.93	1.0E-10	59	intron	rs2288602	8	Yes
	1:236910983	SNP	Het	G/A	50.83	5.0E-05	303	missense	rs80257412	1	Yes
	1:236902594	SNP	Het	C/G	49.1	6.3E-05	387	splice region	rs2288601	8	Yes
	1:236882303	SNP	Hom	T/C	100	2.5E-07	299	synonymous	rs1341864	8	Yes
	1:236883421	SNP	Hom	C/T	100	2.5E-07	301	synonymous	rs1341863	8	Yes
	1:236898942	SNP	Het	G/C	44.86	5.0E-05	185	synonymous	rs2288599	1	Yes
	1:236925844	SNP	Het	G/A	47.51	1.6E-05	402	synonymous	rs12063382	1	Yes
***JPH2***	20:42745062	SNP	Het	C/G	51.85	1.6E-05	542	intron	rs184801349	1	Yes
	20:42814931	SNP	Hom	T/C	100	2.0E-04	68	intron	rs6031442	2	Yes
	20:42747247	SNP	Het	C/T	51.85	6.3E-06	675	missense	rs3810510	6	Yes
	20:42743454	SNP	Het	A/G	61.57	5.0E-05	216	synonymous	rs6093935	2	Yes
	20:42815190	SNP	Het	G/A	48.17	4.0E-05	764	synonymous	rs1883790	8	Yes
***MYBPC3***	11:47361084	SNP	Hom	T/C	99.59	1.3E-06	245	intron	rs2856653	8	Yes
	11:47364248	SNP	Het	C/T	48.39	6.3E-06	839	missense	CM981325	1	Yes
	11:47370041	SNP	Het	T/C	48.2	4.0E-05	639	missense	rs3729989	1	Yes
	11:47354782	SNP	Het	C/T	62.19	2.0E-05	283	stop gained	Novel	2	Yes
	11:47354787	SNP	Het	C/T	50.92	6.3E-05	218	synonymous	rs1052373	8	Yes
***MYH7***	14:23882144	SNP	Hom	T/C	95.41	4.0E-07	283	intron	rs2284651	2	Yes
	14:23883195	SNP	Het	G/A	52.63	3.2E-05	779	intron	rs3729499	1	Yes
	14:23900093	SNP	Hom	C/T	99.66	1.6E-08	594	intron	rs45580436	1	Yes
	14:23902974	SNP	Het	C/A	55.58	2.0E-05	403	intron	rs3729992	1	Yes
	14:23895179	SNP	Het	C/T	47.07	5.0E-06	871	missense	rs121913641	1	Yes
	14:23895180	SNP	Het	G/A	48.93	7.9E-06	750	missense	rs121913637	1	Yes
	14:23902806	SNP	Het	A/G	45.93	2.0E-05	405	missense	Novel	8	No
	14:23892888	SNP	Hom	A/G	100	1.6E-08	582	synonymous	rs7157716	2	Yes
	14:23899060	SNP	Het	G/A	52.54	5.0E-05	788	synonymous	rs735712	1	Yes
	14:23900794	SNP	Het	G/A	45.06	5.0E-05	466	synonymous	rs2069542	1	Yes
	14:23902753	SNP	Het	G/A	34.09	6.3E-06	44	synonymous	rs2069540	4	Yes
***MYOZ2***	4:120079159	SNP	Hom	A/G	98.14	2.0E-07	322	intron	rs11721566	8	Yes
	4:120106982	SNP	Hom	T/C	99.54	1.6E-06	217	intron	rs7661020	7	Yes
	4:120057709	SNP	Het	A/C	46.36	7.9E-05	302	missense	rs76757102	2	Yes
***PRKAG2***	7:151265714	SNP	Hom	C/T	100	2.0E-06	177	intron	rs2241053	5	Yes
	7:151292395	INS	Hom	–/T	84.34	1.0E-10	281	intron	Novel	8	No
	7:151292395	SNP	Het	A/T	30.58	3.2E-05	121	intron	rs35348247	6	Yes
	7:151478406	SNP	Het	C/T	51.55	1.6E-05	419	missense	rs79474211	1	Yes
	7:151483612	SNP	Het	C/T	47.25	4.0E-05	946	missense	rs144857453	1	Yes

^#^*P-value* was generated automatically by the Ion Torrent platform-specific software Torrent Suite v3.6.2, detail refer to Supplementary materials.

## Discussion

This study provides the first comprehensive HCM-specific semiconductor sequencing assay, attempting to facilitate the clinical diagnosis and optimally manage HCM patients. Compared with other NGS platform, semiconductor sequencing has the highest throughput and shortest run time [20,21]. From DNA extraction to data analysis, within only one day, 64.06 kb targeted CDS and flanking regulating regions of 30 genes in up to sixteen samples can be parallelly scanned using one Ion torrent 318-chip. As described in this article, our workflow leads to a mean coverage of 490X, allowing the reliable detection of sequence variants with high accuracy. On the whole, we identified 140 novel sequence variants, which are not listed in the NCBI dbSNP or 1000-Gemome project databases. By bioinformatical prediction of SIFT and Polyphen-2, we revealed potential functional mutations in known disease genes in 104 (87%) of the 120 patients with HCM. This detection rate is in the expected range and provides much better performance compared with previous studies [7,19,25].

To evaluate the capability of our Ion amplicon HCM-specific panel, we carried out assessments of experiments in several aspects. By Sanger sequencing, we dissected the panel technically uncovered 1304 bp regions of the 13 targeted genes in all patients and identified no more potential functional variants. Besides, the panel presented satisfactory results with high sensitivity (100%) and low false-positive-rate (5%) in the following validating tests. Thus, it is reasonable to believe that our panel has enough power to detect potential functional variants in HCM patients.

Ion torrent PGM is considered to have weakness in producing long-homopolymer-associated insertion/deletion errors [21]. Hence, by carefully dissecting the sequences after validation, we found that this kind of primary error type was the most miscall reason in this HCM-panel. Besides, a heterozygous-substitution-miscall (*c.*T136C, *p.*F46L) in gene *MYH7* was detected in 58 of 120 subjects after Sanger sequencing filtration. Since this miscall exists in high proportion of participants and with high coverage, we suspected that it is due to mistake during multiplex PCR. Although this HCM-panel could generate above false positive mistakes, the following Sanger sequencing verification can easily eliminate them.

Although there were some other HCM-relevant genes reported sporadically, such as *TTN*, *MYPN*, *CRYAB*, *MTTL1*, *RAF1* and *FHL1*, the connection between them and HCM pathogenesis were not ascertained [26]. Our panel was designed for clinical genetic diagnosis, hence, selected only causal genes. But we will pay attention to these and other candidate genes constantly, and update our panels once they are ascertained to be pathogenesis in the future.

## Conclusions

This study established a currently most comprehensive and reliable semiconductor HCM-specific resequencing assay and provided a useful, rapid and cost-effective measure for clinical routine genetic diagnosis of HCM. Implementation of this method will significantly improve routine diagnosis of HCM and change understanding of the molecular etiologies of this disease.

## Competing interests

The authors declare that they have no competing interests.

## Authors’ contributions

ZL carried out the molecular genetic studies, participated in the sequence alignment and drafted the manuscript. JH collected data and participated in the statistical analysis. JZ, CC and HW participated in sample collection. HD helped to design the study, DWW (Dao Wu Wang) and DWW (Dao Wen Wang) conceived of the study, participated in its design and coordination and helped to draft the manuscript. All authors read and approved the final manuscript.

## Supplementary Material

Additional file 1: Table S1 Primers used for PCR and sequencing, **Table S2.** Variant Caller Parameter Settings, **Table S3.** Patient Characteristics. Click here for file
