# Supplementary material for: Rapid molecular genetic diagnosis of hypertrophic cardiomyopathy by semiconductor sequencing
Source: J Transl Med. 2014 Jun 17;12:173. doi: 10.1186/1479-5876-12-173 (PMC4072843; doi:10.1186/1479-5876-12-173)
Supplement: Additional file 1: Table S1 — Primers used for PCR and sequencing, Table S2. Variant Caller Parameter Settings, Table S3. Patient Characteristics. [file 1479-5876-12-173-S1.doc]

| **Supplementary Table 1 Primers used for PCR and sequencing** | | | |
| --- | --- | --- | --- |
| **Gene Name** | **Primer Coverage** | **Forward Primer Sequence** | **Reverse Primer Sequence** |
| **Primers for uncovered region by using Sanger sequencing** | | | |
| RYR2 | chr1:237205844-237205859 | CACAGTGCGGAGCAGGGA | GCTCCATCCTCTAGCTTTCCAC |
|  | chr1:237780586-237780599 | AGCCCCGCCCTTGACA | TTTGTCTTCCTGGCTGTGAGTG |
|  | chr1:237796995-237797010 | GATTTTCATTGACTCAGGGACG | ACTTTGCTGATGATGAGTGATGTC |
|  | chr1:237821300-237821300 | AGCTCTTTTGTCTTTACTTTTCTCATA | TTTATAATTTAATGAATAGATCTTTTGAA |
|  | chr1:237823348-237823374 | GCACATTTACTCCCTGAATCTGA | AAGTACTTAAAAATATGGGAGAGGAAT |
| NEXN | chr1:78383424-78383435 | GGTTGTGATGGTCAAATAAAGGG | TTCAGCAAATCTACCCTTCACAG |
|  | chr1:78383810-78383958 | ATGCTTGCTTCTGATGATGAGG | AAAGTGCTGAGATTACAGAGGTGAG |
|  | chr1:78392401-78392577 | GAACAACGACCATCTCTCAAGGA | AGTGATTCTCGTGCCTTAGCCT |
|  | chr1:78395157-78395189 | AAGATGAGGAAAACCAAGACACA | GAGGGAGAAAGGGAGTATGGG |
|  | chr1:78398967-78398989 | GGCTGAGGCAGGAGAATCG | TCTTACCTCTCCCATTTCCTGTCT |
|  | chr1:78401508-78401533 | TTAAGTAGAATGTTTTCAATAATGCCT | TTCAAAGCCAATGTTACTTCTGTT |
| VCL | chr10:75758122-75758133 | CGAGGGAAGCCCCGACT | CGCGAGGCACTCCACGA |
|  | chr10:75802855-75802859 | TCAATGATGAATTTCTCCTATTATGC | AAATCAGCTACTTTTTCTTCCTAACA |
|  | chr10:75834537-75834558 | GGATCTTAAAAGCCCAAAACAT | CAAGTAACCTATGAAACAGAAATGAA |
| MYH6 | chr14:23859266-23859481 | ACTTGAATTAAAGAAAGGAGGAACAT | GAACCACTGCTTGAGAGGAACC |
|  | chr14:23871925-23872009 | AAGCCCAGCACGTCAAAGG | CCATTCACCCCGTCCCTTAC |
| CALR3 | chr19:16590078-16590085 | GGGGGTCACCAGGGGAG | CGCCCAGGATGGAGTGC |
|  | chr19:16593261-16593392 | ATCGCTTGGGCCTAGTTTGA | GCTCCAGAAGCCCCCGTA |
|  | chr19:16593589-16593596 | TCATACTGCGTCAAATAGTCGGT | GGATCGCTTGAACCCAGGA |
| MYL3 | chr3:46899874-46899951 | CTGGAAAGAAGAGGAGAGTGAGTG | GAATACCAGACTTGGGGAGCAG |
| TNNC1 | chr3:52485407-52485543 | GGGTGAAGGTCAGCATCTACTCC | CTGACCCACACTCAAGCCGA |
|  | chr3:52486122-52486131 | AAAGTCCACCGTGCCGC | AGGCTTGGTCCCTCTTGCT |
| PLN | chr6:118880501-118880512 | TCTCTGAAGTTCTGCTACAACCTCT | TCATTTGTGTTATTACTTTGATACTTGG |
|  | chr6:118880715-118880754 | TCTCTGAAGTTCTGCTACAACCTCTA | ATCTCACTGTCACATATTAACCACCA |
|  | chr6:118880861-118880869 | TTTTCAGGTCTTCACCAAGTATCA | TTTTCAGGTCTTCACCAAGTATCA |
|  | chr6:118881188-118881216 | GATCAACAGATGAGAACTGGTGGT | AAGATTTTGCGGACCCACG |
|  | chr6:118881314-118881587 | CTTCCATTCCAGCCTAACATCC | CCACTCCCCACACCCCTAAG |
| FXN | chr9:71650832-71650863 | GGGTCGCCGCAGCAC | AGAAGGGAGTTGCAAGGCC |
| **Primers for evaluating the sensitivity and specificity** | | | |
| MYH7 | Exon 1 | CCCCTGGAACTCAGACCCT | GGAACTCCTGTGGCACATACG |
|  | Exon 2 | CTCGCAGGAGTGAAGAGGATT | TCCAGCACATGGCAGAAACT |
|  | Exon 3 | TCTTGACTCTTGAGCATGGTGCTA | TCTGTCCACCCAGGTGTACAGGTG |
|  | Exon 4 | AGGAAGGAGGGAAAGCCCAGGCTG | TCTGCATGCACTCAATCTGAGTAA |
|  | Exon 5 | ATCTTTCTCTAACTCCCAAAATCA | ACTCACGTGATCAGGATGGACTGG |
|  | Exon 6 | TGTCACCGTCAACCCTTACAAGTG | GAGGCTGAGTCTATGCCTCGGGG |
|  | Exon 7 | CTTGCTGGTCTCCAGTAGTATTGT | CTGCGGTACAGGACCTTGGAGGGC |
|  | Exon 8 | GCCCTCCAAGGTCCTGTACCGCAG | GTCCAAGTCCCAAGGCCAAGGTCA |
|  | Exon 9 | GACAACTCCTCCCGCTTCGTG | AACAGAGGGAGGGAGGGGAGAG |
|  | Exon 10 | CCTTTTGCTTGCTACATTTATCAT | GCCACAAGCAGAGGGGACCAG |
|  | Exon 11 | CTGCTTCCTCAGGCCATGTGCTGT | ACCAATGGCCAGAGTCTTAGCTCT |
|  | Exon 12 | CACAGGGATTAAGGAGACAAGTTT | TTACAGCTGCCCCAAGAATC |
|  | Exon 13 | AGTCATCTCTTTACCAACTTTGCTA | ATTATCATCTGAAGATGGACCCACC |
|  | Exon 14 | CAAGTTCACTCTTCCCAACAACCCT | ATGTGGGAGCGAGTGAGTGATTGTT |
|  | Exon 15 | ACTCACACCCACTTTCTGACTGCTC | GAATTCAGGTGGTAAGGCCAAAGAG |
|  | Exon 16 | ATAACTGTACTCAGAGCTGAGCCTA | TCCATCCCACTGAGTCTGTAAACCT |
|  | Exon 17 | AACTGAGGCAGCCACATTGAA | GTGAAAATGGTCCCGAATGC |
|  | Exon 18 | CATCTCTGTGACTTCTCGAATTCT | CACTGTGGTGGTAGGTAGGGAGAT |
|  | Exon 19 | ACAAAGCCAGGATCAGAACCCAGA | GTCCAGAGTCACCCATGCTCTGCA |
|  | Exon 20 | TGGGTATGAGGGTGCACCAGAGCT | GCATCAGAGGAGTCAATGGAAAAG |
|  | Exon 21 | TAGGCTGTTACCCTTCCTAAGGTA | GCCTCTGACCCTGTGACTGCAGTG |
|  | Exon 22 | GGACCTCAGGTAGGAAGGAGGCAG | TGTGCAGGGAGGTGCAGGGTTGTG |
|  | Exon 23 | TCCTATTTGAGTGATGTGCCTCTC | ATGGTCTGAGAGTCCTGATGAGAC |
|  | Exon 24 | AGATGGCACCAAGCTGGTGACCTT | TCTGGGCACAGATAGACATGGCAT |
|  | Exon 25 | GGCAATCTCACAGTCCCCTAATAA | TTTTTGCCAGGGAGGACCATCTAA |
|  | Exon 26 | ACTCTTTACCTGTATCATTACCAT | GCCTCCATGGACACATAATCAGTT |
| MYBPC3 | Exon 1 | CACACACGGGGCTTGGAG | GGGTGCTTCCTGTCTTGGC |
|  | Exon 2 | GGTAGAGTATTACGTGAGGGGATG | GGGCACAGCCACAGCAAA |
|  | Exon 3 | CAGGCAGCGGGAGGACAG | GCCCTGGACACGCCCTAC |
|  | Exon 4 | CTGAGGCAGGAGAATGGTGTG | GACCACAGGCGGCTTCAG |
|  | Exon 5 | CCTGGAGCCCCCGATGAC | CCCCACACCCCTTGCTTG |
|  | Exon 6 | GCGAAGATCGATGAGCATTTG | CCGAGCCCAGGACAGACAC |
|  | Exon 7 | CTGAATGTTTGGGCGATGG | GCCCCGCAAATCATCCC |
|  | Exon 8-9 | TATCAGCCTTCCGCCGC | TCGACAGACAAGGAAACCAACTC |
|  | Exon 10 | TCTGAGTTGGTTTCCTTGTCTGT | GGGCCTTTACTTCCTCCCTATT |
|  | Exon 11 | GGGGTGAGTCTCTGTGCCTT | CGTCCTCCTCTGCTGGTGC |
|  | Exon 12 | AAGGGGCAGAGGGACAGG | GGACTCCGCTCTTTCCATGTA |
|  | Exon 13 | GGAGCAGGGTGCGGGC | GCCGTTGAAGTGTTCCCGAC |
|  | Exon 14 | GAGGTCAAATGGCTCAAGAATG | TCCCCAGGTTCCCACATC |
|  | Exon 15 | GGGGGGCACAGGGATTAT | GCTGCCCCACCATCACC |
|  | Exon 16 | GGGTGGCGAGAAGTGTAGCA | AGGTGGTGTCTCTGCCCGT |
|  | Exon 17 | TGAGTGTGAAGTATCGGAGGAGG | AGGCTGTCAAAGGCCCAAG |
|  | Exon 18 | CACCACCCCTCAGACACTTG | GTCTCTGACGAACTCTCCTATGTGT |
|  | Exon 19 | TAAAAATCAGAATACCAACAAGCCA | TGCGGGAAAGTGAGCAGAAC |
|  | Exon 20 | CCCTGGCTGGGGTATCTG | GGTGGCACTATCAGGGTTCC |
|  | Exon 21 | CCTGTGCTCCTCCTGGCTC | GGGGTGTGCAGCAGGTCC |
|  | Exon 22 | CAGGCAAGGTGGGCAGTGT | AAGGGCTCGGCACCACG |
|  | Exon 23 | GTGCCACAGAGATGATTTTGAAC | CTGTCCTGTTGCCGCTCG |
|  | Exon 24 | GCAGCCTGTGGCGGTTAG | TCAGCCGCATCCACCG |
|  | Exon 25 | AGTACAGTGGGAGCCGCCT | GATGAAAGGAGACTGTGGATGTGA |
|  | Exon 26 | TGGCTGCTGTTGCTGTTTGT | TGGGTGTCCTCAACTTTCGG |
|  | Exon 27 | CCTGGGAGACAAGAGCGAAAC | ACGATGGCTCCAACCCCT |
|  | Exon 28 | CCCTGCCTTTTGAAGTGGTG | CCCTCTTTGGTCCAGGTCAC |
|  | Exon 29 | GCCTGTGAACCTTCTCATCCCT | CCCACCCCTGCCAACCA |
|  | Exon 30 | CCCCTTGTTTCCCTTCCC | TCCTGATGCCGAGAGCCT |
|  | Exon 31 | CAGAAAGCCGACAAGAAGACC | ATAGGTGATGCCTGTTGGTGAC |
|  | Exon 32 | ACCAAGGAGCCCGTCTTTATC | CCAAGGTGGAGAGAAAGCAGG |
|  | Exon 33 | CCTGCTTTCTCTCCACCTTG | CCTGGTCACTGAGGCACTG |
| ACTC1 | Exon 1 | CCCCTGTCCAGTCCCCAC | CTTGCCTTGGCTTGGATTTG |
|  | Exon 2 | TCGTATTGCTTGTTATTTCTGGTATT | CTAGGGAATGGGAGGAAAGG |
|  | Exon 3 | ATGGTCATTTGAAAGTGTCCTCG | AGCTACAGAAATAAAGAGTATCACAGTCA |
|  | Exon 4 | GAGCGTGGCTACTCCTTTGTC | TGTGTGGGAATCCAAACTATGAC |
|  | Exon 5 | AGCTCCCCCACACAAAGAAGT | GTAGGAGACAAGAAACTGAGTATGAGG |
|  | Exon 6 | TTTTCTGTGAATCCTCCCAATG | TGCAAGTCCTGGTCTGGTTTAT |
| PRKAG2 | Exon 1 | CCCTGCTCACCCTCCCTC | TCCCTCTACCCTTTCCCCAA |
|  | Exon 2 | GGGGCTGGGTGTAGCAAAC | GGATGGGCTCGGTTACGG |
|  | Exon 3 | CAGTGGCTACCTCTGTGGAA | AACAGATACAGGCACTCAGCA |
|  | Exon 4 | CCTCTGACTGCCCCCGA | ATTTCCATTTGCTATTGTGCC |
|  | Exon 5 | GGCGGGTGGGGGCAG | GCTTCCGCAGGACGCAG |
|  | Exon 6 | CCTGGCCCTTGATCATACTG | CTCAGCACAGTGCCCGATAG |
|  | Exon 7 | GCACATGCCTGTCATCCCA | CACCCTGCCAGCAAGAATG |
|  | Exon 8 | GTTAGTTCCGTAGTACTTACTGTCAGGT | ATCACCATCAGCACACCATACC |
|  | Exon 9 | CGCACCTGGCCGCCT | CTGCTGAAGGTGTCCTATACATGTC |
|  | Exon 10 | TGATTAACACAGGAGTTTTAGGCA | GCGTACAGTGTAGCTGGAATTAAG |
|  | Exon 11 | GTTAACATAGATTGTGAAAAATGAAAAA | GAGAGAATGCCGAGTTTTGTTG |
|  | Exon 12 | ACAAACTGGTTCAAACAAAAGATAC | CGTGCCCCTTGGTGCTG |
|  | Exon 13 | CCAAGGGGCACGCAGG | AATTCTGATAATATCCTCACACCCC |
|  | Exon 14 | ATAACAGTAGCAATGGCATGTCGTA | GGAATCCCATCGACTGAACC |
|  | Exon 15 | CCAGCCACTGTCTAATGTTTTTGTA | CAACTGAAGAGAAAACGAAAATGG |
|  | Exon 16 | CCCCTCCTCCGAAGCCTC | TTCAACATCACTGGAAGAAATACCT |
| MYOZ2 | Exon 1 | CAGATAAGGAATGCGTATTGAAGT | TTAGTTACCATAGGCTTTTCTACACC |
|  | Exon 2 | AATGCTTACCTTGGGATTTTTACTC | ATGCTTCTTTTGTTTACCCACCT |
|  | Exon 3 | CCTATTATTGTGCTTACATTTTTGAGTT | CCCACCTCAGTCTCCCAAAGT |
|  | Exon 4 | GGAGGTTAGTAACTCTCGGGCA | AAAATGACCAAGTTTGAAAATAATAGTAAT |
|  | Exon 5 | TGAGCTCAGTGTACAGGGAATAGAT | AATGTCAGAAAAGGAAAATCACTAAAT |
| ACTN2 | Exon 1 | GGGTCCGTTTGCCAGTCAG | GCACAGCACCTCCACAAAGC |
|  | Exon 2 | GCAGCCCCAGCCTTGTAGAT | CAATAATACCATGTTCTTGCCCAG |
|  | Exon 3 | GTTCCCTCATTGCTGTGATTTAGA | CGCCCTAAGAGAGAGCCTGATAC |
|  | Exon 4 | CGGGTGGTTTTCACTGATAATACAT | GAAACAGTGCTGCCGAGGTCT |
|  | Exon 5 | TTGAAGTCCCTAAGTGTCATCAGAAT | CCCCCAAACTCAGTAGCATCC |
|  | Exon 6 | CAAGGAAGTCAAAAGGAGAGGAGT | AAATCAAGCCAAATCATCAAACAT |
|  | Exon 7 | GTGTGTGGGTGGGAAGGTGTC | TTTGCTGGCTTGTTTGAGGGT |
|  | Exon 8 | ACACTCAGAAAGATTCCAAGGTAAGA | GTTCTCCCTGGTTTCTCTCCTTC |
|  | Exon 9 | CACCGTCTACAGCACGCCAC | TTTTTTCACCCTCTGTGCCTCA |
|  | Exon 10 | GTGTTTGGTATCGTTGGGGTG | ACATGATGGTTGGGAGAAAAGTATTA |
|  | Exon 11 | TGAGGTGGGGGGTAAGGAAG | AAGAGCAGAGAAAGGTGACGACA |
|  | Exon 12 | GTTCTTTGGAATCTCACCGCTC | GTGCTGCTGGATGGTAGGACA |
|  | Exon 13 | CTATCAGCCCACTCTCTATGTCCA | TCCCATTTCCCTCTTTCTTCC |
|  | Exon 14 | TGAGCATCGGGTAATCTTTTGTAG | ACTTGATTGATTTTAACGGGTTTG |
|  | Exon 15 | GACATCCTTTTTCCATTTTCCAG | ATAATCTCTGCTTTCTTTCCCCAC |
|  | Exon 16 | CCCTTGGACTATTCCCGCAT | CTGACTGAGTGGGTGGCTGG |
|  | Exon 17 | CTTCCTCTGAGAAAAGTGAACCGA | CCACAAATAGTGACTGGAAAGGCT |
|  | Exon 18 | CAAGTGTGGGGAAGGCTATCAT | AGTTTTGGTGGCTATTTTCTTTTGT |
|  | Exon 19 | TTGAGTGTGTGGTCTCCTTTCG | GCTGGGATTACAGGCGTGAG |
|  | Exon 20 | TGAAGGGAAACGCAGGTAATAAT | AAGCACGAATGCCAGGAATG |
|  | Exon 21 | GGCTTTCTGTTGGTTGTCTGGT | TTGCTGGCAATTACTGAACTACG |
| JPH2 | Exon 1-1 | CTTTCTTTCCAGCAGGCAGC | CCATCAGCATAGGTCTCGGTG |
|  | Exon 1-2 | TTGTCAGGGGCTATGATGAGATG | CAAAACCACACAGCAAATCACTTC |
|  | Exon 2-1 | CTGGCTCACCCACTCGTGTTAT | GCTGGCTACCCACGGACG |
|  | Exon 2-2 | CGTCGTGCCGCCTACTGA | CCCGAGCGTTTGTCGTTCT |
|  | Exon 2-3 | GCACAGCAACGGCACGGT | AGCAGAATCAGGCTTGGAGACAG |
|  | Exon 2-4 | GCAGCCGTGTCAGCTTCCT | GCAGAATCAGGCTTGGAGACAG |
|  | Exon 3 | CCTGCGTGAAAGAATGCTGTG | ACTCTGCTGCCCTCCCCTC |
|  | Exon 4-1 | TCAGTGCTATGTGAGTGCCGAC | AGCTGTGGTAGCCCTGGTAAAG |
|  | Exon 4-2 | CCAGCCTCCCTGTGTTGC | TCGGCTTTGGGGATGATG |
|  | Exon 4-3 | CGCTTTACCAGGGCTACCAC | GAACCCGACCTTAGGCACAC |
|  | Exon 4-4 | CGCTTTACCAGGGCTACCAC | CCCAGCAACCCCCAAATC |
|  | Exon 5 | CAGGTGACAGGATACAGCAGTGA | AATCACACTCTAGGTCTTGGCTTCT |
| **Primers for Semiconductor sequencing** | | | |
| CSRP3 | chr11:19207718-19207940 | GGTGGAGATGAGCAAATTCTGTCT | TGCCAAGGGAAATCTACGTTTCATT |
|  | chr11:19204152-19204369 | ATCTGTGCAGGATTACTTGGCA | GCTCAGACAGAAAGCCCTGAAAA |
|  | chr11:19209762-19209890 | GGCCATATCTGCGCCCATAG | TTTCAATTCCAATCCCATGACCCTT |
|  | chr11:19213826-19214047 | TGAATTTTGTGATGCTGTCCGGAT | GGTATTTGCTTTATGTCCCCTTAGACT |
|  | chr11:19206422-19206646 | AGGCATGAACGTAATTTCCTCTCC | AAACAGAATGTGTTGCCTACCTCAT |
|  | chr11:19209580-19209803 | AGTCCATTCTCCCACTTCCAGAA | CGGAGATCTACTGCAAGGTGTG |
| FXN | chr9:71687511-71687720 | TTTGTTTTGTCTTCCAGTGGACCT | TTGGCCTGATAGCTTTTAATGTCCTTAA |
|  | chr9:71679841-71680016 | CCTAATCCCCTAGAGTGGTGTCT | CACAATGTCACATTTCGGAAGTCTTTAA |
|  | chr9:71661205-71661428 | GGCACTCGAATGTAGAAGTAGCA | ACCTATGACGTGGAAGGTGTTTTATC |
|  | chr9:71668097-71668259 | CGCTGGACTCTTTAGCAGAGTTT | CTCTGCTGGAGGGAAATTCTTCATT |
|  | chr9:71650555-71650748 | CGAATAGTGCTAAGCTGGGAAGT | CTGGGTGACGCCAGGAG |
|  | chr9:71679664-71679887 | CTGATTCTGAAAGCAAAGCTCTTCC | GATCTCCACCCAGTTTGACAGTTA |
|  | chr9:71714721-71714944 | GGTTAGTGCCTCTTCAGTTCGAG | CTTCGTTGCTCACTTGCTGATT |
|  | chr9:71650691-71650849 | GGAGCAGCATGTGGACTCTC | TGCAGGTCGCATCGATGT |
|  | chr9:71687381-71687555 | ATAAGAAGGCAGATATACACTAGCTCAT | CCCAGTCCAGTCATAACGCTT |
|  | chr9:71668011-71668141 | GAAGCATTTGGTAATCATGTTTTGGGT | GCTTGTCTGCAAGGTCTTCAAA |
| GLA | chrX:100662769-100662921 | GGTAGGCGTCCTTGCCAATC | GGAAATTTATGCTGTCCGGTCAC |
|  | chrX:100656604-100656825 | TGGAATGAAACATTACCATCTGCCA | GTGACTCTTTTCCTCCCTCTCATTTC |
|  | chrX:100652749-100652972 | GGAGAAAAAGGTGGACAGGAAGTAG | TGGGTAAAGGAGTGGCCTGTA |
|  | chrX:100653255-100653457 | ACTGATAGTAACATCAAGAGCAAGGGA | CCGACACATCAGCCCTCAAG |
|  | chrX:100655586-100655810 | TGGTTTCCTTTGTTGTCAAGTTCTATCT | ATATATATAGCCCCAGCTGGAAATTCA |
|  | chrX:100658874-100659035 | GGGAGCCATCCAACAGTCATC | AATGGGAGGTACCTAAGTGTTCATTT |
|  | chrX:100653621-100653842 | CCCATATGGAGAAACCACTTTCCA | CTGGACATCTTTTAACCAGGAGAGAATT |
|  | chrX:100656456-100656656 | TTAATGAACTGAAAGAGAAGAGATGGGA | GGTTGTTACTGTGACAGTTTGGAAAATT |
|  | chrX:100662610-100662809 | CAACTGTTCCCGTTGAGACTCT | GGGCTAGAGCACTGGACAATG |
|  | chrX:100652931-100653117 | GTGTGATGAAGCAGGCAGGAT | CTAAGCAACCACACTTTCTTGGT |
|  | chrX:100658720-100658918 | AGGGCTGTTTCTAAACAAGCTTCT | GCAGGTTATGAGTACCTCTGCATT |
|  | chrX:100653795-100654008 | CCTGGTCCAGCAACATCAAC | CCTCCTTTAAACTGTTTTCATCTCACAA |
|  | chrX:100653414-100653630 | CCTTATCCTGAAGGAGAGCTTTGG | TCCATATGGGTCATCTAGGTAACTTTAA |
| MYH7 | chr14:23884740-23884889 | AGCAGGAAAAGCATTGAGCATCTA | GGAGCAGGAGCTGATTGAGAC |
|  | chr14:23892699-23892894 | GCTCTGGGCACAGATAGACAT | GATCATTGCCAAGCTGACCAAG |
|  | chr14:23894445-23894665 | GCCTGGGCCTCAGAGAAG | CCCTCCCTAGTCATGGCCAA |
|  | chr14:23902679-23902827 | CTCTCTGTCCACCCAGGTGTA | GTGCCTGATGACAAACAGGAGT |
|  | chr14:23886372-23886594 | TTTGTTCTCCCGCTTGAAGGT | CCTGAGACAGACCCTGGACA |
|  | chr14:23898382-23898606 | CCACCATGCCAGTCTCCCTA | GGCCAGCAGTCATCTCTTTACC |
|  | chr14:23893043-23893261 | GCCTGGGTCAAGGTCAGTATG | TGGAGGATGAGGAGGAGATGAATG |
|  | chr14:23901611-23901825 | GGCCTGGGACACCTGATG | TCCTGAAGGGAACTGGGATAGG |
|  | chr14:23888650-23888859 | GAGACTGTGGTGGGAACCAT | CGCCCCCAACATCCATCATA |
|  | chr14:23902237-23902447 | GGACATGGATGGAGCAAGAACA | GGCTCTTGCAGACAGTGACC |
|  | chr14:23896322-23896546 | ATGGTCCCGAATGCACCAA | TGCTTCTTTTGTTGACTCTCCTTCC |
|  | chr14:23897790-23897963 | CTCCTGCTCCAGCACAAAC | GGCTCAGGCACAGTGGAC |
|  | chr14:23884320-23884522 | GCTTCTGCAGCTGCTTCTTG | GAGGACTTGACCAGACCATGTG |
|  | chr14:23891327-23891551 | GGGTCTGCTTGTACTGTTATGGG | CTCCTTCTCTCTACCAGCTGGAA |
|  | chr14:23893942-23894097 | AGGGTGGAAGAGCCAACAGTA | GAGGAGTTCACACGCCTCAA |
|  | chr14:23885192-23885384 | CAGTCCCCTCTGGGTGAGT | GGACTCGCTGCAGACCTC |
|  | chr14:23886022-23886237 | GACCAAAAGCCTGGAGCTCA | GCAGCCCTCAACCGAGTTA |
|  | chr14:23883163-23883370 | GGACGAGCTCTCCTATGCC | GGGATGCTACCTTCTATGACTGT |
|  | chr14:23899016-23899184 | CGCTGCTTCAGCTTGAACTTC | GGATCTCACTTACCCATCATACTTCTTT |
|  | chr14:23900725-23900949 | GGAAGGCTCATATCTGAGACCATT | GGTCCTGTACCGCAGAAAGG |
|  | chr14:23896906-23897130 | GCCAATGATGTTGTAGTCCACGAT | CAGTGATGCTCTCTCCTGCTTC |
|  | chr14:23881947-23882171 | TCTCGGCTTCAAGGAAAATTGCT | CATCAGACCCCTCTCACCTTT |
|  | chr14:23900062-23900265 | CCAGGTTGCCATGGAGATAGT | CCTTGTGCCCAAACCCTAACTT |
|  | chr14:23886753-23886959 | CAGCATTGGAGCGCTCTAC | GTTTCCTCTTGTCCCCATCCAC |
|  | chr14:23895132-23895329 | CTCCCCCTGTTCTATGAGCTCT | GTGTTCTCACAGACTCCTCCTACT |
|  | chr14:23887445-23887654 | CTGAATGGCGTCCGTCTCA | GAAAGCTGAACCCACCTCCT |
|  | chr14:23889249-23889464 | GTCCCGCCGCATCTTCT | GAGCCCTTTGTGTCTGACCA |
|  | chr14:23884852-23885062 | GCTGCACCCGCTCACTA | GCCCACTCTCCTGATCCTCA |
|  | chr14:23892850-23893060 | GCCTCTTGCAGAGCTTTCTTCTC | ACTGACCTTGACCCAGGCTA |
|  | chr14:23902785-23902998 | ACACGATCTTGGCCTTGACAA | AAGGGAAGAATGGGCAGATGG |
|  | chr14:23882913-23883135 | TGTCTGGGTATGCCTGCTG | CCCCCTCTCACCTCATGCT |
|  | chr14:23900556-23900766 | GAGAGGTCAAGACCAGATGGTCTA | GACTTGGACTGGTGGAGGAATG |
|  | chr14:23900914-23901133 | AGAGCTCTTCTCCCTCCCTTTC | GCTTGCTGGTCTCCAGTAGTAT |
|  | chr14:23888323-23888545 | TTTGATGCAAGGCTAGTCAGTGT | GGGATAGAGAGGAGTGCTGATCT |
|  | chr14:23893217-23893420 | GCTTCTTGGCAGTGAGCTCAG | TCTACCTGCAAGAATGAGGACCTTA |
|  | chr14:23884544-23884767 | CGGAGAGACACTGGTCTGGAT | TGCATAGATGCTCAATGCTTTTCCT |
|  | chr14:23902407-23902532 | CATCACCTGGTCCTCCTTCAC | GAGCACTATTGCCCTGTCACT |
|  | chr14:23897636-23897830 | GGGCTGCTATTTTGTCTATGGT | AGCAGTTCTTCAACCACCACAT |
|  | chr14:23884145-23884363 | GCTGGTTGTCACTGTGGCTAT | CGAGCAGATCGCCCTCAAG |
|  | chr14:23901812-23902036 | AGTTCCCTTCAGGAAGACCCTT | CTGGCAAGTCACTGCTCCTT |
|  | chr14:23894058-23894266 | CGGACTTCTCTAGCGCCTCT | AGGCTCAGCACTCCTTTCAATG |
|  | chr14:23891505-23891636 | CTTCTTCTCTTGCTCCAGGGAT | GTCCTGAACACAAAGATTTACCAAGTC |
|  | chr14:23888947-23889155 | AGAGGAAGGGAAGTGGGAAAATG | AACCTGCAGCGGGTGAA |
|  | chr14:23885348-23885563 | GCGTGTCTCTGCGTCCAG | CCTGACTGTCTGCCTGCAT |
|  | chr14:23895912-23896131 | GAGATGTCCTAGGAGGTCCTGTT | TTCCTGCATCTCTTTCTGGCAT |
|  | chr14:23898128-23898349 | GTGATTGTTCTCCCACTCCCA | ACCCTGCTCAATATGGGTCTCT |
|  | chr14:23898915-23899059 | GAGATGACTGCTGAGCAGACA | GCCATCATGCACTTTGGAAACAT |
|  | chr14:23894851-23895073 | GAGTCAATGGAAAAGAGATGTCTTCCT | CAGATCACTGCAGAGCATGG |
|  | chr14:23896748-23896954 | GCAGAATCCCTGCTCCTCTGTA | CTTCTCCCTGATCCACTATGCC |
|  | chr14:23890079-23890301 | GCTGTGAACAGGACACCCTAGA | GCCCCACGAGTCTCCCTTA |
|  | chr14:23887259-23887483 | GCTTCCCTGAGAGGAGAAGGA | GCCCAGTGGAGGACCAAGTA |
|  | chr14:23899710-23899933 | CCCTGTTTGCCCCTCACTG | CGAGCAGCCTCCATGAGA |
|  | chr14:23886209-23886420 | GGGAACACGGTAACTCGGT | CTATGAGGAGTCCCTGGAACATCT |
|  | chr14:23886583-23886800 | GTCTGTCTCAGGACCTGCCT | AGAATGAGATCGAGGACTTGATGGT |
| MYLK2 | chr20:30419477-30419696 | CCCTGCCCCCTGCTATC | TTGCACACATACAGTTCAGGTTCT |
|  | chr20:30418650-30418853 | CCGGGCATTTGGTGAAGATCA | TCCCAAAGTTCACCTTCAGCTTC |
|  | chr20:30407307-30407523 | CCTGACACACTCCACTCTTGTT | AGAACTCCCACTGAGGAACCT |
|  | chr20:30411155-30411376 | CCCCAGGTATCACTTGGTGAA | GCCTCCTTGGAGTTCATACTGA |
|  | chr20:30414352-30414575 | TGGGTGGCAGGGAGTGA | CTTGCTCCGTGCCTGGT |
|  | chr20:30407997-30408217 | CCAAAGAAAGCTCCGGATCCA | TCAGCCTTGGGCTTCTTGAC |
|  | chr20:30408301-30408428 | GCTCACCTGCCTTTCTGCATAG | CCTAAGACAAGGGAATTAAGCAGGA |
|  | chr20:30409354-30409491 | GGAAAGAACATCCTGGCAGAGAG | CCCCACCTCGGATTTCTCTGA |
|  | chr20:30411970-30412161 | GGGTCTGGGACCAAGTTAGGAT | CACTACCTTGTCTTTGGGAGTCTG |
|  | chr20:30414609-30414812 | CGGAGAGCTCTTCGAGAGGAT | CTGGTGACAGCACTGATCTCA |
|  | chr20:30419838-30419992 | CATCCCTGGCTCAACAACCT | AGGAGCTCTTCCCACTAGCAA |
|  | chr20:30418570-30418747 | AGGGAGGGTGAGCTGGT | TGTCCCCTGCCAACTCAGA |
|  | chr20:30418810-30419027 | CCCCAGGTATAACCCCAACGA | AAGACCCTAGGGCCAATCTTG |
|  | chr20:30411334-30411508 | CCGGGAATGTCAGCAGTGAAT | CATGTCCATGTGGCCATGTCT |
|  | chr20:30414428-30414652 | TGAGGTCATGAACCAGCTGAAC | TCAGATGGTAGTCCTCATCCACA |
|  | chr20:30407885-30408043 | GGAAAGGAGGGTGGATCCTGAT | GCTTTGGCATCTTTCTTCAGGGT |
|  | chr20:30408174-30408360 | CAGACTGCGACACCTGAGA | GGGATATTCACCTGGAGATGATGG |
|  | chr20:30409215-30409400 | CTTCACCTCTGTGTTCTCACCTT | GTTTTCTCTCCCACTTCCTTCTGG |
|  | chr20:30409448-30409635 | GGATTGAGTTCCAGGCTGTTCC | GTTCAGACCTCCGAATAGGTAGACT |
|  | chr20:30421448-30421672 | GGCTGCTCAGACACCCT | GGCCTTCTGGGCTGTGT |
|  | chr20:30412116-30412277 | GGCAGCCAAGGTCATCAAGAAA | ACTGCAACAAACTCCCATACCAG |
|  | chr20:30419666-30419882 | TCACCCCAGAACCTGAACTGTAT | GTTACAGCGTTTGGCTTTCTCC |
| RYR2 | chr1:237947965-237948135 | TCGTCGAAGGTGCTAAAAAGATCAA | GGTCACTTTCCTCTGTCAGCTC |
|  | chr1:237863493-237863672 | TTGGAAGATACGAGTCCTCCCTTA | GTAACCCCTTTGGGCTGGT |
|  | chr1:237951335-237951498 | AACAAAAGTTGAGGCAGCTTCAC | TGAGCCTAAATTATTACGGTATTGCACT |
|  | chr1:237205650-237205864 | GCCTCCTCCTCCGCTCT | GCAGGAACTGGATCTCGTCTT |
|  | chr1:237889418-237889640 | ACAAGTTGCCTCGTGAAAATTTCTAAAC | GTAATCTCTAGGCCATTGAGCACT |
|  | chr1:237955257-237955480 | TCGTTTTTAGCCTTTGCTATTAGTCCTT | GGCTTGTCACTTTGGCATTTTCA |
|  | chr1:237920992-237921216 | CTTAGGTGAAACTGGACCAATGGTAG | TGCTCTCTCTTCAGTACTGTGGTAAA |
|  | chr1:237802166-237802370 | GCTAGCAATTAGGATTACATTTTGGCT | AGGCCTCCGAATGAGCAATC |
|  | chr1:237632228-237632428 | TGTCTCAGTGTTGTATATGTACGTGT | TGAGCACAGTTTTTACGATTTCCTCTAA |
|  | chr1:237823207-237823378 | GCACTTAAACACCTGCATATCTACAATC | TTACCTTTTCAGACAATAGCTTATATGGCTT |
|  | chr1:237617717-237617935 | CGAAGGCTTCCACAGTCGAT | ACCAAGAAGCTGTTTTTACAAGAGAATA |
|  | chr1:237875061-237875187 | AGCAGAACTTCGTTGTACAGAATGAAA | AAGTCACATTCAGATATTTTCCAGAACAGT |
|  | chr1:237865207-237865414 | GCTTAAGCTTTCTCAGGACAATGTA | AGTTATAAAACTTGAAGCAGCAATTAATACAA |
|  | chr1:237958459-237958677 | TTCATTGTAAGTTTACGTGGCAGGATAT | TAAACTGTGCTTTAAGAAAAATGTAGAGGATTC |
|  | chr1:237956992-237957213 | ACCTTCTAGATGCTTCTTCCTGCT | AATTTCCGTGCCACTTCCTTTTC |
|  | chr1:237818930-237819151 | GCTGCAGCTTCTTTTCTGTTTGAT | GTGCCAGTTTGAAAAGTTCTTGTTCATA |
|  | chr1:237608651-237608861 | TTTGGAAAAGAGACGTTGGGAGTAA | TGAAGTATCAAACAACTACGCCTAC |
|  | chr1:237841266-237841490 | GCTTTCTAACCTTTGCATTATGGATGAA | GAAAAGGGCAACGATCTAATGAGTA |
|  | chr1:237995786-237995985 | TCTACCTTATGTTTTGTTAGCACACACT | GGTAGGGTTTTGGTTTTTAGAGGTGA |
|  | chr1:237821193-237821324 | TCCTGTAAATTCTGTGAAAATCAAGCTCT | ACCTTGTCCATTGACCATTTGTCAT |
|  | chr1:237872732-237872921 | AACTTTAAGGAGTAGCTGAGAAACCAC | GTAAGCAGGTGCCTACAAACAGTA |
|  | chr1:237806620-237806834 | TGTATAGTTGATTCATGCCGGGAAG | TTGGGATGTTGAGTACGGAAAACA |
|  | chr1:237732332-237732553 | CTCCTACTGTTGTGTTGATAGAAGGG | TCAGTGTGAACATCATGGTGTGT |
|  | chr1:237551346-237551527 | ACACTGACAGTCCAGACCTGA | GGCAAAAGAGTGCAAGTCTCAATG |
|  | chr1:237777780-237777999 | GGACATCCTCAAGTCCAAAACCA | CGGAGTGGCAGCTTCTTTAAAC |
|  | chr1:237659877-237660086 | ATTGATGGTGGACCACACAGAG | TGGAACACGGTGAAAGAAAATGGATA |
|  | chr1:237777475-237777678 | CCTCAGCTCCTCTATGCCATTG | CCCTGGAAGGCCGTGTTTTT |
|  | chr1:237895279-237895503 | AGTGCTTTTGGATTTGAGTGAACATTT | ACGGGTGTCTTTATTTGCTCCT |
|  | chr1:237936852-237936979 | TGGGAAAGATGTTATTGATGAACAAGGA | CTACAGATAGCAGCCATGTTTAAATGTT |
|  | chr1:237817565-237817689 | CCCACATAGCTGCTGACAAATCATTA | CCCCAGAACAACTTTCTTGATAAATGAA |
|  | chr1:237924189-237924387 | GCTCTCCTGAGCAAAGAAAATGTTCTA | TGACTCGTTTCAGGGAGGGAA |
|  | chr1:237586303-237586429 | TGACTGTTCATTGTCCGACATATGTTT | GGAATGGCTGTCCCCATCTTATG |
|  | chr1:237774149-237774331 | CTGTCACACGTCCTGTGGAG | CCAAGCGTCCCAATCAGAATG |
|  | chr1:237890387-237890604 | GTGGAACATCCTCAGAGATCTAAAAAGG | CCTTCTCTTGGACTATTGTACCCCTAT |
|  | chr1:237791189-237791351 | CATGTTTGTGTTGCTCCATCGG | TTCTTTGCCCATTCTCACACTCA |
|  | chr1:237540545-237540769 | GTTGGGAATCATTGGCAAAGAAAATAGA | TCAGCAGATACATGATCCTACTTTCCA |
|  | chr1:237813137-237813341 | CCAAACACAAATGGCCTTCTACTC | ACAGCCCTTAGAAAGTCTATACACAGTA |
|  | chr1:237729814-237729993 | TCTCTTTCACCTCCCCATCCAA | CTCATGTCTCCAGCAGTGACC |
|  | chr1:237758790-237758993 | AAATTCAGCATCAAACGCAGCA | CTCCCTAGAAGACTGATATCATCACTGA |
|  | chr1:237969511-237969722 | CCTTCTCGACATTGCTATGGGATTC | GTTTAGAACAGGTGCAGACCCT |
|  | chr1:237824194-237824376 | GGAGACAGCATGGCCCTTTA | TCTCCCTTCTAAATTTTGTGACTCTTCAG |
|  | chr1:237755061-237755185 | CCAGATTACAGCACAAGCCATT | AGAAATAAACGACAAAAGCTGCAACC |
|  | chr1:237664079-237664257 | TGTTTAGATCTGAGTGCCCCAAG | TGGAGGTATGAGAAATGAGAAAGGAAAG |
|  | chr1:237935235-237935459 | TTTGCCTTTGGAGTTCTTAGAATGACT | CCGTATTGTTTCAACATACCAACCC |
|  | chr1:237752980-237753164 | CCTAGGGTGACAGCTCTCACA | AGGTGCTGACATCCTTTCCAAAG |
|  | chr1:237788957-237789128 | CTAGGAATTGAGCTGGATGAAGATGG | CCAGTGCACTCTCATACTGCTTA |
|  | chr1:237433755-237433974 | CACGTCTCACTTATTTTTCCCTCTCTTT | CTTCAATGCCACTAGTAAAACTTGCAT |
|  | chr1:237604508-237604728 | TCAGAGGGCTGAATTTTTCAAGGAA | AGCCATAGGCCTGTGTCTACA |
|  | chr1:237804148-237804346 | TGTCAGTTATCCCATTTGCTTTCTCTT | ACAGAGAAATCAGGATTGCTGCT |
|  | chr1:237947006-237947221 | GATGGTGGATATGCTTGTGGAATCT | GCACAAGACAAAAGAAATTCCGTTTC |
|  | chr1:237765332-237765550 | CGAGTACAAAATTATTTCCTGCGGTTT | AAGCATGGCTATCTTTTCTTGAAAGAAT |
|  | chr1:237947340-237947551 | CCCAACGATACCCGACTTCAG | CCTTCGTTGACCACGTCAAAT |
|  | chr1:237947626-237947850 | AGATCTCGGAGTCGGACTTGA | CCATGTCCTTCACGGTCATCTTTTT |
|  | chr1:237814680-237814901 | GGCATTATGAACATTAGCTTTGTTCCA | AGCGTCAAGCATGATGTATCTAAGAAAT |
|  | chr1:237780577-237780705 | GTCTCTTAGATGTGCCTACTGCT | GTTGTATCGGAAACGTTGATTGTCTTG |
|  | chr1:237794681-237794870 | AGATGTTGCTCTGAGCTTTACCC | AATAGCATCTCAGGAATCAAAGACGTT |
|  | chr1:237670013-237670203 | GTGTTGCCTCCTCATCTAGAAAGAAT | GTCACCCCATAGCGGGAAAA |
|  | chr1:237993763-237993985 | GCGAGTTGTGTTTTCCTTTTGTTTTG | AGGGACCAGTGGCGTCTA |
|  | chr1:237850683-237850893 | TTAATTTCCCCCTGTCTTTTCTACCTTC | ATGCAACATAGCAGCACACAAC |
|  | chr1:237693658-237693882 | GCTGCTGTCTTGGGTTATTCTGG | TCCATCAAGAGTTATTTGGTTGCACTA |
|  | chr1:237756784-237757008 | CTCAGTGAGAATCTTTCCTGGACAA | GGAAAAGGAATAGCTAGGTTCACCAATA |
|  | chr1:237837355-237837557 | TCAAATTACTATGCTGTGTTCTTGTCCT | GTGAAAAGAGTGTGTACTTAGGGTATGT |
|  | chr1:237965066-237965284 | CATTGAATGTGAAGGCCGTCAG | TCTGCAGAACTCCCACAGTCTTA |
|  | chr1:237753831-237754011 | TGTGGACCGCATTTGGGATT | CTGAGAACCAAAAGACTTCTGAGTGA |
|  | chr1:237754129-237754351 | GACTTGGAAGATTATGATGCTGATTCTGA | GGACTGTAACCTTCCTCACATAACTAC |
|  | chr1:237949194-237949416 | GGTCACATGGCTCCCTCAAT | GCTCCCAAAGACAGAGTACAAGAAAA |
|  | chr1:237519159-237519383 | TGTGGTGCAAGGACCAAATTGA | GTACAAGCCACACTAGGAAGCA |
|  | chr1:237881711-237881859 | ACAGTTGATGCAATAGAAGACTCCTTTT | AGGTAGTAAATGTGGCTTAAGAATAAAATTTG |
|  | chr1:237872185-237872345 | TGAAACCTCAGCTCTTGAAAACTCATT | GGCCAGTGTGGTGAACTCATC |
|  | chr1:237905512-237905646 | GCTCTTCTATGTCTAATGTTCTTTTGCAC | AAAACTCTTCACTTCCTCTTCACCAT |
|  | chr1:237831073-237831254 | AGCACTAATACTGTATGAGTTATTCCTTTAC | GGACTCCAACTCCATTTTCTTTTTCTTT |
|  | chr1:237656220-237656424 | GGGAGGATCAGCTGAAAGTAATTTCT | TGGTACCTGTCAGGTGACAT |
|  | chr1:237666606-237666828 | GAGGGCGACATGGAGAATTCA | GCCATTCGAGTGGATCTTGGTA |
|  | chr1:237711716-237711918 | GTCTCTGATTTGTAGTTACCAGCTGA | CTCAGACCAAATGCTAATTCCCATGTA |
|  | chr1:237798132-237798347 | GGAGTTTATAGTTACAGCACGATCCA | AAAACCGTGAAAAAGCATAATTCAGACA |
|  | chr1:237787028-237787252 | CCTCCTTCTTCCTCTTTCTTGTTTTTCA | CGCATATACACTTAATTTCGTCTCAGGA |
|  | chr1:237532738-237532959 | CGTTCCTTTGAGAGCAAGAGAGT | CCCAAATGGATCAGCATGACCA |
|  | chr1:237982299-237982521 | TGTCCCTCCAAGAAGTGATACCA | TCATCAGCTTCAGTGAGGAGGAT |
|  | chr1:237944792-237945013 | TGTGAGAATAAGTATGATGTTCCTGCTG | CCATAAGCCCATGCTGATCCTTT |
|  | chr1:237655004-237655226 | ACATGTTGTACTGAATGCACCTATGTA | ACCTTGTGATTTCTTCCATGTTTGTCT |
|  | chr1:237919523-237919746 | TGGTCTGTAGAAATCTGTTGTGCTTAC | GAGAGCAACGAGGAAATCTGAAGA |
|  | chr1:237870132-237870313 | GCATGCCACGTCTTGCTAAA | CATGAGTTTCTCCAAAGACGGTATGT |
|  | chr1:237870374-237870597 | GAAGTCATACTGCCCATGCTTTG | GGGTACAATGTCTTCTTCCAGTTCTTA |
|  | chr1:237205840-237206032 | GGCGAAGACGAGATCCAGTT | ACCTGCACACACTTGGTGAT |
|  | chr1:237756608-237756830 | GTAGTGTTAGTCCTTTGTGCCTACA | CCACCCAGACATTAGCAGGTTC |
|  | chr1:237527631-237527845 | TCCTCTCTTTTCCTTATGCCCCTA | ACCATACAGAAAACATACAAAGTACCACAT |
|  | chr1:237666427-237666650 | GCCCTTGAATTCTAAGGTGATCACATT | CATACCCAGGTGGAGGAAGAAATT |
|  | chr1:237765177-237765379 | ATTCTTGAAGGAGCACATCAATCGATAT | TGGGACTTGTAGCTTGTGCAA |
|  | chr1:237669860-237670066 | AATGTCGTGAAATAGATGGCTTAGAACT | ATGGATATTCTCTGCCAGTTTTTCTCTT |
|  | chr1:237758627-237758833 | GCAGTCAATGGAGTTTTGCTCAGA | CCCGCACATACCATATAGCAGT |
|  | chr1:237890223-237890441 | TGATAATCTTAAGGTACTTTTGGAATTGGAA | GCTTGGACAGTAGTTTATGCCATACAG |
|  | chr1:237632379-237632528 | TCTTCTTTTGCAGCGGCTCTAA | GGAGTGATCTATCACAACAAGGAAAAA |
|  | chr1:237806445-237806668 | TGTTTGAGCAAGTAAATATTCGCAGGTA | GGACCTAATTCTGATGGCTTCTCC |
|  | chr1:237655104-237655274 | AGGCATTCTGGAAGTTTTACACTGT | ATCTAAAAGGATTTGAGAATTCATTCAGGGAA |
|  | chr1:237796799-237797020 | TGATTGAGACCTCAACGTATGAAC | ATTTACTTACCAAGACCAACACTGCT |
|  | chr1:237494140-237494360 | AGATGATGCTGCTGACTGCTC | GACACCTGTGAAAGAAATTGTTCCTATA |
|  | chr1:237817409-237817617 | AGTATACACGGTGATAGTATTCTCCAA | AGGCAGTAATATTTCCAGCATCTTTCA |
|  | chr1:237872102-237872236 | CATCATGTTAATGAATGGTTTTACAGAGC | TTGAGTTTCTCCATTAACGGCAAGA |
|  | chr1:237948091-237948301 | CCGAGGATCTGACCGACTTAAAG | AATGCTGCTGTGACTTAGAGATAAATT |
|  | chr1:237801602-237801823 | CATCTAATGAGTTTTCAGCCAAGGGATA | ACTCAAAAGACCTCAAGAAATCAAGTCC |
|  | chr1:237659778-237659921 | CTTTCCTTACATGTGATTCCCGTCT | GTTGCTTCAGCTGTCACAAAGG |
|  | chr1:237732508-237732669 | GGGTGTATGGTTGACATGAACGA | GCTTCCTTGTGCTGCAAACTTAAAAA |
|  | chr1:237936747-237936904 | GTCCAAGACTGGTTAGTCAATGGT | GGATAGCTTTGGAGAAATTCCGTTG |
|  | chr1:237674915-237675132 | CCACACAGTTGAATTCCATTGCT | GGGAAGGCAGGTTGGAAAATAGA |
|  | chr1:237868471-237868681 | TCACTTGGAAGACCTTAAAACATAAGCA | CTTCATATCTCAAAGCACTGCACA |
|  | chr1:237817641-237817830 | GTGCTGCCTCAGAAGAAGAAC | GCCATACGTGTCTTGGTTAGCAAA |
|  | chr1:237777305-237777519 | TCTAATCTTACTACCACTCTCCTCCCT | CAAACCAGGCATGTACTTGTTCT |
|  | chr1:237991619-237991841 | GCCAAGGAACTGAATTATTCATTGCT | CCTCCTTCCCACATTCATGATTCTATTG |
|  | chr1:237777957-237778175 | GCAGTTGATTGAGCCCAGTGT | TGAAATTCACTCAAGTCTCTTGTTCTGA |
|  | chr1:237819100-237819311 | TTGGTCCTCCATTTTTCCCAGAAA | ATCTGCTGCAATAGAAAGAGTCCATAC |
|  | chr1:237777635-237777828 | TCACCCTGTTCCCTGATGAGAA | TTTAACAGCTTCTGTCAGCATCTGTA |
|  | chr1:237773991-237774192 | TTCTAGTCATTACTTTGTGAACCCCAAG | TTCAAAAACTGGTTGGGCATTCTG |
|  | chr1:237586386-237586607 | CATTAGGTGGAGTGGAAGCCA | ATCAAAGGAAATATGACCGGGTAACAT |
|  | chr1:237791023-237791230 | GGGTACAGGATATGGACTTGAAATG | CCCCCAATGCCGTCATACT |
|  | chr1:237791298-237791422 | CTGGCATCCCTTGGTCAGATT | AACATTTACGAGACAAAGGGTTGTAGT |
|  | chr1:237957165-237957370 | AGGTCCCATTGGTTATTTTTAAGCGA | CAGGAACTCCAACCAAATATTTTCATGT |
|  | chr1:237813287-237813424 | CGCTTCTCTCATTGACTCATTACTTCA | CCGGAATAGAATTTGTTATCCACTCAC |
|  | chr1:237729947-237730160 | GGACGGTGGTATTTTGAATTTGAGAC | GTACTGTGAAGCTACCAAGAGGAA |
|  | chr1:237954683-237954904 | AAGCCTGTTGATTCAGTGACCT | GCAGTAGTAGATACGTTCATCGTGAAAT |
|  | chr1:237922983-237923192 | TTCTAGAATGGAAAGCCTGTTTTGGT | ACCACTGAGTAGAACCCCCAA |
|  | chr1:237969390-237969563 | GCGTTCTGTTTTCTGACATGTTCTT | CTGAGGACAAGATGGTTCTTAATGTCTT |
|  | chr1:237862227-237862412 | CTCAGGGCAATTTATACAGCATTTTGT | CAGACACTGGGTCCTTCTTAATATTCTT |
|  | chr1:237886387-237886608 | ATTCCCATCTTCCCATTGTAACCTTT | TGTATGTGGCTAAGGCAAATATGATTGA |
|  | chr1:237824017-237824235 | AAGTGAAGGCAGTCTATTTTAGAGCAAA | AGAAATACGACGAGTCCGGTTG |
|  | chr1:237754942-237755104 | GCCCAGGTTTAAGGCAACTCAA | ACATCTTCGGTGAGTCTTGCAG |
|  | chr1:237580250-237580474 | CCATTTGCTTTTCTCTCCTAATTAGCTT | AGCTATTTGGTACACTTGGTGCAT |
|  | chr1:237802328-237802529 | GGAAAATGCAAATGTCGTGGTGA | CTCGTGAGGGTGTGTATATTAGACCTA |
|  | chr1:237663904-237664127 | GTGCATTGGTTTGTCACTACTTATTCAA | GTTGTCCATTAATTCGGAACGAGATG |
|  | chr1:237893526-237893700 | ACACCCTTTTTCTGAAATTGTGCTTAC | AAGCCTAGACCCTCTTTCATCTTAGA |
|  | chr1:237753121-237753345 | GGCTCAAGTGGGTAGGATGAA | TCCTGCTTTTTAGAAATGGCCTGAT |
|  | chr1:237880468-237880692 | CAGATCCCAGCACTTCTCTTTGTT | CGCTGGGATGAAAAGGAGACT |
|  | chr1:237811712-237811936 | CATTGACACCAAAATTCACTTCTCTCTT | GAAGTCCATCGCTCCAATCTCA |
|  | chr1:237821273-237821400 | TCATTAACAAATATGCAGAACACTCCCA | CTGTCATGGTCAGAAATATTTGAACAGT |
|  | chr1:237604680-237604864 | ACGGTGACTCAGTATGCTATATACAACA | ATCAGCCAATTATTTTTGACTTGTGCTT |
|  | chr1:237961294-237961518 | TTAATGGTTGAAGCCAACAAAATGCT | TTCCCCTTTCAATCTAAGTCTGTAGGA |
|  | chr1:237972158-237972382 | TCCTACATTTCTAATACCTGGTCCTTGT | AGCAGGAGACATTTTTGTTTATGTGAGA |
|  | chr1:237946852-237947054 | AGTGAGAGATAAACAGGGACATATCCT | GAGAATCATCTCCACGTTGTTGGA |
|  | chr1:237947174-237947385 | CCATAAGCACTACACGCAGTCA | CGCTCTCTGCTAATTCCAGAAAAGT |
|  | chr1:237947505-237947668 | GTCAAGGAGTCCAAAAGACAGTTCAT | TCCTTATTCGCTGACCTCTCGT |
|  | chr1:237920859-237921042 | AAGTCAGTCTCCCTATGCCATTA | GCAATTCCAAGTTTCAGAGTAGCTG |
|  | chr1:237947797-237948012 | AGAGCCTGAAGAAGCAGATGAAAAA | TGTTGGCTAACAGTTCTGCAACT |
|  | chr1:237780651-237780854 | CTTTTCAGATGATTTTGTGGCTAAGCTC | GCAAGGCAGAGATCCTGAATGAC |
|  | chr1:237550526-237550749 | CATGTTACAGCTTACCAGAGCCT | TCACAATTCCAAAGATGACTGTCAACT |
|  | chr1:237955437-237955650 | GCTCCCCACGAGAAGTTCAAG | AGGTACCTTGGTGCCTTACTATCTTA |
|  | chr1:237537967-237538144 | AACAGACGAACAAAACCTCTACTTACA | CCTGCAGGGTTCTCTCTGAAC |
|  | chr1:237617553-237617759 | GGAGCAGGAGGACCTTTCTGA | AGGCTTACGGACTCTATAGGCAA |
|  | chr1:237753959-237754182 | CCATAGACAGTTCCCCATGTTTAAAGG | AGCTGTCTTCATCAGAACCTCAAAG |
|  | chr1:237843706-237843928 | CCCCTGAAAGATTCCACTACGTAGA | CTGCATATACACCTAAGTCATACAACAGT |
|  | chr1:237872303-237872445 | GGAGGCAGAACTCCTCATCCTA | ACGTTATGACCGAATTGGAGAAATTACT |
|  | chr1:237837963-237838185 | ACACACAATTTTACCTTCTAAGAGGCT | CAAACACTGGGCTATTAGCAATGG |
|  | chr1:237905593-237905740 | CAGAGTTGTCATGATGAGGAAGATGA | CCATGAAGCAAGAAACATAAATGTCAGT |
|  | chr1:237874954-237875138 | TCTCTGTAAAAACTTGCAGGTTCTGT | CCTTTGACATCTTTGACTTGGTATCAGT |
|  | chr1:237831190-237831318 | CAGAAATGATGGCTGAAAACTACCATAA | CATACACTCATGGAATTGAGCTCAGAT |
|  | chr1:237619847-237620070 | GAATGACAGTTTTGGATGTCTGATTGTG | AACTCTGTTGCTATGAACCCAATCAT |
|  | chr1:237896957-237897181 | GCCTAGCCTTCTGCTTAAACACT | CGGAGATTCATGCTACAAACAAAAAGTT |
|  | chr1:237994771-237994949 | TTTCCATACCGTTCATTTCTGATCAGTT | ACTGTTTAATGCACACATTTTGTTCTGA |
|  | chr1:237829766-237829972 | CACAAGGTTTTTAATGAGGCACTGT | GCCAAATATATGCAGGAAAGCATTACAT |
|  | chr1:237934067-237934260 | GGGTGGCTGGTAATGTTTGAT | GGCTCTTGTTATTACTGGCTTGAAAAC |
|  | chr1:237870261-237870418 | CAACTAATGTGGAAGATGTTTGTCCAA | CCACCAACGAGACATGTAGCTG |
|  | chr1:237863634-237863857 | CCAGTTCACTCACACCCGAA | GTAACCCAGATGTGGCTATTACTGT |
|  | chr1:237951189-237951380 | GATGGCCTAAAATATTTGTTCACTTGGG | TCTGGTTCTCCGTATCTGTGTGT |
|  | chr1:237713818-237714034 | CGGCTGCACCCTGTGTT | CATCATCTTGCAACCGAAGAAGAG |
|  | chr1:237941892-237942116 | CCCCTGTTATTGTACAGTTTGTTTTGG | CTCCAGTTTGTCATTTAAGAGAGAGGAA |
|  | chr1:237711569-237711761 | GCTAAAGTGAAAGGAACCTAGGCT | GGCAGGCTTGTATCCACTTG |
|  | chr1:237788793-237789010 | CAGTGAATGTTATTTATGAGGGCTGGTA | TGTTAAATCACTGTTTCCATCCAGAGAC |
| ACTC1 | chr15:35082573-35082795 | CGTCATCCTGACTGGAAGGTAGA | CTTCAGAGTTCACTGGAAGTTTTTGTTT |
|  | chr15:35084334-35084540 | CTCATTGCCAATAGTGATGACTTGG | CTCCCTATAATTGACTTCTTGCTTCAGA |
|  | chr15:35083360-35083576 | CATACGATCAGCAATACCAGGGTA | CCTTGACCTGAATGCACTGTGAT |
|  | chr15:35085393-35085540 | TCGGTGACTTGGGAATGTGATT | GATGACTCAGATCATGTTTGAGACCTT |
|  | chr15:35085662-35085872 | TGGTGATGATACCATGCTCGATG | AAATATGTTCCTTGACTTGGGCAGTTA |
|  | chr15:35084160-35084381 | TTGAGCTGTGTGGGAATCCAA | AAGAGCTATGAACTGCCTGATGG |
|  | chr15:35083194-35083407 | GCTAACAGGTAAAGTTTGTAGGAGACAA | GTCTTATCTGGAGGCACCACTATG |
|  | chr15:35086815-35087038 | GCCATTTCCTAGATCGCTGGA | CCTGCAGAACCCCCTGAA |
|  | chr15:35085492-35085708 | CACGTACATGGCAGGGACATT | AGGCATCCTGACCCTGAAGTAT |
|  | chr15:35084581-35084805 | CTGTGGCAGATGAGACACACA | GAATTTACCTTGTTCTTGTCTACTTCCC |
| ACTN2 | chr1:236883402-236883580 | CAGAAATTGTTGATGGCAACGTGAA | CTGTTGGACTTTAAAACATCTTGCTCAA |
|  | chr1:236902564-236902773 | CTGGTGTCTTCAGCAGTATTTTTGTG | TCTGCAGCGTGTTGAAGTTGA |
|  | chr1:236912429-236912603 | GGAGAAATTGCTAGAAACCATTGATCAG | TTCTAATGCATAGGGCATACAGAGTTG |
|  | chr1:236924283-236924499 | CGTGTACATGTTTCTTTGCCACTT | GCAGAAGGAAATCTGTCAATGCAG |
|  | chr1:236882141-236882360 | GCATCAAGAGGTCACTGTCTCTA | GCTATTAGGTCAGTGTGGCAGA |
|  | chr1:236849989-236850208 | CCCGGCGTGCAGTACAA | TCCACAAAGCGCCCTCTC |
|  | chr1:236920747-236920969 | AGAGACTTAGAACTGATCTTTCCCCTT | CCCCAGTATTGCCCTTCAAATAAGTA |
|  | chr1:236918261-236918428 | TCCCTTAGAGGGCACTTCACT | AGCTTGTCGATGTTGTTCTTATAGTTGA |
|  | chr1:236898870-236899089 | CCTTCGTCTGTATGTGTGTGGT | CAAGTATGCACACACAACTAAAACCAA |
|  | chr1:236906153-236906377 | TGCTTGTTACACATTTGCTTCCC | TTTCAAAAATCCAATCTGACTCCTGTCT |
|  | chr1:236910900-236911112 | TTAGCCCATGTTCATTTTCTCTCATCA | AAAGATTTCATGTCAACAAGTGGCTTC |
|  | chr1:236890914-236891137 | CAGATGCTGGCTGCTTCTTTC | GGCTAGTGCTGATGTGCTCATTT |
|  | chr1:236914833-236915011 | TCCATCATGGCCATCCAGAAC | CCTTCGACGTCAGCTTCAAGAAT |
|  | chr1:236912279-236912478 | GAGCATCGGGTAATCTTTTGTAGACA | TCTTGGCAAACTCCAGGTGAAG |
|  | chr1:236889155-236889379 | CTGTTTCAAAAGTGACTAGGAGCTAAGT | CCCCATGCTACCCACAGATTC |
|  | chr1:236902733-236902898 | GGAGAAATGCCAGCTGGAGA | CAAACGCCTTTAGGCTAGAAGGA |
|  | chr1:236894439-236894660 | TGTCCGGCCTAAAGTGAGGTA | AGTCTTCAAATTTCCTGTAACTCAGCAA |
|  | chr1:236849895-236850027 | CCTCCGTGGGTCCGTTTG | CTCATCCTCGTCGTACACGTAG |
|  | chr1:236917209-236917426 | GCTGGCCTCTAACCCTTGTTG | TTATCGCAAGGCTTGTGAACAG |
|  | chr1:236900340-236900556 | CTCTCATCACCCACCTCGTT | GCGTGAGGATGGACAGAACT |
|  | chr1:236918377-236918573 | TGAAGCAGTATGAGCACAACATCA | ATGAAGAAAGCACCCTGAGGAG |
|  | chr1:236907879-236908101 | CGCTGTGCCCTCAGCAT | GAGGACGGAGGGCATCTG |
|  | chr1:236881107-236881327 | GATCTGAAATCCGATGGACCTGT | GATGCTCACCTACTGATAACACATGAAA |
|  | chr1:236914654-236914876 | CTCCAGAGACTCTTGCAATCATCA | CTCTGAATCACCTTCTCCACCTC |
|  | chr1:236925728-236925952 | ACTGACTGCAAACACGTGTGTA | TCTGATGGGATGAGTGATTACAGAA |
|  | chr1:236922952-236923176 | GTTCAGTTGGTCAAGTAGAGTTGCT | CCTTGGTTTGAGCTTGTCATCTTTT |
|  | chr1:236883251-236883449 | CCAGACATACTGCGGTGCTT | TCCAGATCATACCCAGGGTCATT |
| ANKRD1 | chr10:92678875-92679083 | AAAAAGCCCTTACAATGATTTCAGGTTC | ACCAGAATGCTTGTCAGTAGAACTC |
|  | chr10:92675894-92676100 | GGGTGAATGAAGGGAGAAGGAG | TGCCCTAGCAACCAGGTTCTA |
|  | chr10:92675215-92675439 | ATTGTGGGAGTCGTTTCACCTATTT | GCCTCAGACCCACCTTGAAC |
|  | chr10:92680655-92680878 | GAGTTCCCTTGCATTACACCTGAA | GCTATATAAGCTGACCGGTGTGG |
|  | chr10:92678586-92678794 | TGAGCTGGATTTTGCAGTGCT | AGCTCACGGTAACTCTCATGCTATAT |
|  | chr10:92678801-92678930 | CATTCAATCAAACCCTCCACAGATATTT | AACTAAAGTTCCAGTTGTAAAGGAACCA |
|  | chr10:92672569-92672788 | CCATGCCTTCAAAATGCCAGT | GAATTTACAGATGTCACCCACCTGAA |
|  | chr10:92677445-92677665 | GTCTACTTCTTTTGGCTCATTCCCA | AGCTTCATAGCTCTTAAAAGGGTTTACA |
|  | chr10:92679904-92680128 | GGATTCCACAGATGGCTCTCA | GCTGCCATCTTCTTCCCTCATAG |
|  | chr10:92675475-92675689 | GGGAGTGATTCCAAATACAAAAGCA | ACAGCCTCTGTCCTCATTCCTA |
| CALR3 | chr19:16594739-16594963 | ACCTGGGCTTTGTTGTCTTTAGT | GATTTAATTGTTACGCTTTTCCCCCTAA |
|  | chr19:16606517-16606705 | GGAAATGTAACATTAGGCTCAATTTGGT | CCATTATCACCTCTAACCGGTGTT |
|  | chr19:16593455-16593613 | GGCATCCCTGGCATCATACA | CTTACATGTCTTTGAAGGACTGGGA |
|  | chr19:16591393-16591615 | GATGTAGGAAGAGTTAATCACGTGCT | CCTGAGCTTCATGTATTTCCAAAGGAA |
|  | chr19:16601035-16601243 | TCAATACTACTACAAACCTTGGATCCGA | AGGTCTTTCCTGCAGACATTGAC |
|  | chr19:16589817-16589989 | ACCCAGAAGCATGAAACACTGT | AGGCACGAACATTACTTCAATCAATTTC |
|  | chr19:16606841-16607041 | CCCCTTCACCTCCGTCTAGAA | GAGTTGTGACGGGAGTTGTAGT |
|  | chr19:16593240-16593439 | GTAAAGTTAAAACCAAATGACCTGCCA | CGCTGCCCATGTCATTGAGA |
|  | chr19:16601196-16601420 | GCGATTTTCCATTCAGGTTCTTCTG | CGTCATTCCTGATCCTTCATGAGTATAT |
|  | chr19:16589935-16590100 | GGGATCACTAAAGTTCATTCCTTCTGT | TCTTTCTTTCTCCAGGGTCCAGA |
|  | chr19:16595918-16596127 | CTAAACTTAGTCTGGCAGGAGTTAACA | GGTTATTCTACGGTGATGCATTATGGAA |
|  | chr19:16606676-16606886 | GGAAATAACACCGGTTAGAGGTGATA | GCTACCGTCTATTTCCAAGAGGAAT |
|  | chr19:16594564-16594784 | TTACGATACCTTCAGATGTGTAGAGATG | CAGAATCGAAGGATTGGGAACAG |
| CASQ2 | chr1:116269433-116269645 | TCAGAAGTAATAACATCCCTGCTAGTCA | GGAGCTGGTGGAGTTTGTGAAG |
|  | chr1:116275437-116275647 | AAAGAAAAGAAAGAGTGGTGAGAGTTCA | TCTGTGTTCATCTTGAAAATGTCTCTCC |
|  | chr1:116260395-116260609 | GGGCTGGCAAAGGTGAGAAAT | CTATTCCCATTCCAGCTTCTGAGTC |
|  | chr1:116245460-116245681 | GATGCTCTCACAATCTAAGCTGTGT | CGGGTGGGTCCTTAACAGAGA |
|  | chr1:116310903-116311073 | AGACAGCATGCCCTTTGGTT | GAAGGACCGAGTGGTAAGTCTT |
|  | chr1:116243794-116243990 | CACCTTGTGCTGTCTGTATGGT | CTGGAGGACTGGATTGAGGATG |
|  | chr1:116283281-116283497 | GGGTTCTATCTCTCCCTATATTTGAGG | TTCCTACGTCAGGATGACCTTCT |
|  | chr1:116287376-116287583 | GCAACTGTACTTTTCCTTTTGCAAGA | CTACCATCTTTCTGCCCTTAGCA |
|  | chr1:116280782-116281006 | CTGACATACCTTGTTTGGAATCTAGCTT | GAAGGAAATCTCTTGTTTGAACATGTGT |
|  | chr1:116247752-116247976 | TGAACGCAATCATGAGGTTGTGA | GCTCTCCACATTAGAAGCTGTGTATG |
|  | chr1:116269603-116269809 | TCTCAGGCACCTTTGGTGTTC | TGGACAACACACTGCTATTCTAGACTA |
|  | chr1:116268018-116268239 | CCACCTCAGCCAAATAAACTTGGT | TGTCTGCCACAGTGATCCCTA |
|  | chr1:116311030-116311199 | CCTGCTTGAAGTTCTTCTCGGA | CAGCATATTTGGGAACGAGAAACAA |
|  | chr1:116243941-116244076 | TCTTCAGTGTTTATCTTTCCAGAAAGCA | CCACTCATTCATTTTGTCCCTCACT |
| CAV3 | chr3:8775518-8775728 | GCCACACAGCTCGGATCTC | GTCTCGCAAACCTGACACTCT |
|  | chr3:8787279-8787462 | GCTACACCACCTTCACTGTCT | GAGTAGATGTGGCTGATGCACT |
|  | chr3:8787173-8787323 | GCTTCTGTGAGTTGAGGCTTCC | ACAGACGGTAGCACCAGTACTT |
|  | chr3:8787415-8787634 | CATTAAGAGCTACCTGATCGAGATCC | GCAGCCCCTGTGAAGAAGTC |
| JPH2 | chr20:42743442-42743615 | CAGGAGGTGAACAAAGAGGATGG | CATCCAGAAGTTCCTGCTCTGT |
|  | chr20:42788341-42788557 | GACCTTGTTGCTCTTGAGCTG | CTACATGGGCGAGTGGAAGA |
|  | chr20:42788662-42788823 | CGAGCTGAGGTCGCTCTTA | CTCAGCCTCCTGGCCAA |
|  | chr20:42788906-42789043 | GTGCCGTTGCTGTGCTC | GTACCAAGGCCAGTTCACCAA |
|  | chr20:42815043-42815240 | GCTTGAGCTCTGCCGGATT | GGCGAATACTCTGGCTCCTG |
|  | chr20:42747093-42747314 | GCCCACCCTAGGGATAGCC | AGGCTGGTTCTCTGGAAGGAT |
|  | chr20:42744733-42744902 | CGGACGGAGTGACTGACC | GCTGCACGAGCGTGAGA |
|  | chr20:42744413-42744627 | CCTGGGCTCGGCTTTGG | ACCAGGGCTACCACAGCTAT |
|  | chr20:42743294-42743490 | GGTGGGCCTCAATTAGTCACA | GGTGATCCTGCTGAACATCGG |
|  | chr20:42788173-42788393 | GAGACAGGGCCTCCTAGGTT | TGCTGGTCAAGGACACCAAG |
|  | chr20:42788521-42788698 | GCCCGAGCGTTTGTCGT | CAGCCGTGTCAGCTTCCT |
|  | chr20:42788805-42789015 | CATTGGCCAGGAGGCTGA | CGCCATGGCTACGGAGTA |
|  | chr20:42814860-42815080 | GCCCATCTGATTCTGTGCCAAT | GCTTCAAGGGACGCTACGG |
|  | chr20:42815200-42815422 | GCCACCTCAAAGCCAAAGTTC | GAGGAGTGTTGCAGGCATCA |
|  | chr20:42744966-42745129 | AGCAGGCTCTCCGAGTTCT | CGACTGAGCCCATGAATGAGG |
|  | chr20:42744287-42744449 | CTTGCCCGACAGCCTCA | GGAGCCCAAGCCCATCATC |
|  | chr20:42744594-42744808 | GTGGTGCGCACAGCATAG | AAGGACGGCCTGCTGAG |
| LAMP2 | chrX:119565032-119565211 | AAAGACTGATCTCAAAATGCTGGGAT | CAAGCACCATCATGCTGGATATG |
|  | chrX:119572825-119573048 | GGAAAAACCAAAGTCAGGCAACTAA | TGGCAGAAGAAAAAGTTATGCTGGATA |
|  | chrX:119602878-119603099 | CGGACCAGTCTTTCAGGTTGTA | CGAGCCTTTTCCCTGGTGTT |
|  | chrX:119582750-119582887 | GACTTCAATGAAAGCTACCTGGTATACT | TCCAACACTACTGGGATGTTCTTG |
|  | chrX:119575455-119575668 | GTTGCACAATTTGGTCAAGGCTAT | TTTCAGTGTCTGGAGCATTTCAGATAAA |
|  | chrX:119590386-119590577 | CCCAGAGTTTCAGCATTAAGTCAGTC | AATGCCACTTGCCTTTATGCAAAA |
|  | chrX:119581623-119581779 | GGCTGAAGGTAGGATACGCAAT | GCTGGAACCTATTCAGTTAATAATGGCA |
|  | chrX:119576399-119576579 | TCTAATCCTTCAGGGTAATCCACAGT | CGTATCTGTTATCTGTAGACAGTTGTGA |
|  | chrX:119589092-119589305 | AGACATGAAGTTTCCATGCTAGTTAGG | TTTACCAAGGCAGCATCTACTTATTCAA |
|  | chrX:119562245-119562467 | CTTAAACAATCTATTGCACAGCATGTCC | GCTGACTCTGACCTCAACTTTCT |
|  | chrX:119580086-119580309 | CCCCATGCAACTATTTAGACTTTCAGA | CCTTTTCCCTTTCTTCTTCTCCTGA |
|  | chrX:119565161-119565346 | CAATCAGGTTGCAGATTCTAAAATTGCT | GTTCTCAATTGTCCTTTCTCCACATCTA |
|  | chrX:119572993-119573216 | CACGTATTGATTAGTGTTACAGAGTCTGA | TCTTTGACTTCGAGACATTCTTTTGTGA |
|  | chrX:119582839-119583025 | GTGCCATTTTGGACAAAAGCTTGTA | CCTACCTTCTTGGAAATAGCCATTGTT |
|  | chrX:119590530-119590704 | GCGTACTGTGAAATTCATCTGCCA | AGTGGTGGGTAGAGCTTGTTTTTAA |
|  | chrX:119575616-119575814 | AGGCTGAACCCTTAGATCAAAGGTA | GCCAACTTTCCCATTTTTGATGTTGA |
|  | chrX:119581729-119581920 | GGTAGCCAGCAGACAAGTATCAT | GTCCGCTTTGGTTTCTTTTGTCC |
|  | chrX:119589253-119589464 | TTGTAGGAAAATGAGACGCTGTCAA | CCTGTTTGTTTCTCACCCCCAA |
|  | chrX:119562423-119562582 | CACACCCACTGCAACAGGAATA | GCTATGTACACTTTCAAAATGCATGTTT |
| LDB3 | chr10:88492585-88492805 | TGAAATCTGCTCATGCCCTGT | AGCCTCCCGAATCCTTTGTTG |
|  | chr10:88451630-88451844 | CACGGCCTCTCTCTGCAT | CACCCTGAGAGCCTGCAC |
|  | chr10:88458931-88459093 | GTGAACACATTCCCTAACCCCTT | GGTGCCAGTTGCGCAATTT |
|  | chr10:88446756-88446971 | CCATGTAACCGCCACCTGTT | CCTGGGAATACATGCTGATGGG |
|  | chr10:88466248-88466468 | GCTCCCTTGACCTGTTGTCTTTT | GTCGGCAGGACTTGAAGCA |
|  | chr10:88469621-88469838 | GGCTGTCCTTCTGGGTGTA | CGACAGCCTCCCCTCCT |
|  | chr10:88477674-88477839 | CAAGTTCTGGGAGCTGCCTTA | CACAGTAAACGTTGTTCTGCTCTTC |
|  | chr10:88441133-88441342 | GACGCGTGTGGCCTCTAA | CTCGGCGAGTGAGGAGAAG |
|  | chr10:88441443-88441585 | AGCCGAGGCAATATAACAACCC | GGAGAGCTGTATGTCCGTTACCTA |
|  | chr10:88478442-88478666 | CCCTCTCTCCTTTCTGTCCTGA | CTGTGCCTGCCCCTTTCT |
|  | chr10:88439072-88439296 | TCCTCAGGACCACCTTCCTT | CTCTGGAGAGCTCCCACCT |
|  | chr10:88476068-88476274 | TTTTTGGCTTTTGCAGTGCCT | CGAGGGTGCAGGGTTATAGTTG |
|  | chr10:88476268-88476396 | ACCCTCGGTGGCCTACA | GGCAGGGTCTGCTTGCT |
|  | chr10:88428382-88428606 | CCTGAGTGCCCTCTCACT | CACACATGCCCTCCTCCAA |
|  | chr10:88459055-88459243 | GTAACAGCCCACGTTTTGCC | AGCAGAGTTATACATTGTCAAGCATCA |
|  | chr10:88439735-88439957 | TGACTCTGGCTCTCTCTTGCT | GAACCAGGGCTGAGTGGAC |
|  | chr10:88446928-88447118 | CATCCATGCGCAGTACAACAC | GGAAGAAAGCTGGTCCCGAT |
|  | chr10:88466429-88466585 | CTCCGCCTCCACCACAG | CCTGCGGGCCCTAACTA |
|  | chr10:88452209-88452426 | GGGACTCAGTGCCCACAG | CTTGAGAGGTCCCTTCCATGAG |
|  | chr10:88485867-88486050 | GGGTCTTCACTCTGCTTTTCAT | GCCTCTTCTCAGAAAGCCAGA |
|  | chr10:88477794-88477939 | CTGGCAGATGTGTGCTTTGTG | GAGACATGGGTCAGAGGAAATGG |
|  | chr10:88441291-88441484 | CACCTTTAGCCCTGCCTTCTC | CTGCCGAGTACAGGCCAAT |
|  | chr10:88475894-88476109 | CTGTGAACACCCTGCTGAGAA | GGACGGGCTGTAGGTAGATG |
|  | chr10:88476118-88476342 | CAATTACAGTCCCACTCCCTACAC | GAGAAGCTATCATCTGTCACCCA |
|  | chr10:88476360-88476547 | GCAAGAGCACCACCTCCATC | GGGCACAGCTGGACCATAC |
|  | chr10:88445327-88445550 | TGGTTCTGCCTCCACAATGAC | GCCAGCAGGCACACTCTATC |
| LMO4 | chr1:87805171-87805377 | TTTGGTTTAGGATTATTCACTGGTGTCA | CCCATGTAGGTAGGAAAGGTATGATTTT |
|  | chr1:87805766-87805936 | CCGGGAGATCGGTTTCACT | AACCTTCCAACTTGCTCTAATAAAGCTTA |
|  | chr1:87797615-87797833 | TGCTCTCTCTCCGGCGATTA | GCTGTGCCAATAGCTGTCCAT |
|  | chr1:87805609-87805813 | CGCTAAACCCTAAGTGTCTCTGT | ATGTTCACAAAATAAACTGCCATTGATGT |
|  | chr1:87797790-87797964 | CGGACCGCTTTCTGCTCTATG | CCATCTCCAGGAAAGACAGCAA |
|  | chr1:87810387-87810573 | GGGAAGACAAAAGCAGTCATGT | CTACAAAGGTGACTTGTCTACTGAAGA |
| MYBPC3 | chr11:47368124-47368254 | GGCTCCTGGCAGAATTAGGG | AGGTGGCCATACCTCTCATGT |
|  | chr11:47372009-47372215 | GCCCTACCCACGGATCCT | GGGTCTCTGGTTAGTGGCT |
|  | chr11:47359195-47359413 | ATCTGTTTGGCGCCCTCA | GGACGAGCAACGTTACTCAAG |
|  | chr11:47353303-47353497 | CCCCTACAGCCTCCCATTTAC | GCCCAGATGGGCAATAGCTTC |
|  | chr11:47364895-47365093 | GCAGGAGCAAAAGGATGGGAAATTA | CGCTGAGGTCAAATGGCTCAA |
|  | chr11:47358929-47359126 | CAGGCAGGCTCACCGATAG | CATCCTGGAGCGCAAGAAGAA |
|  | chr11:47364326-47364520 | GGCCTCTTCTGGGCAGATG | GGAGCCAACCCTCATGCT |
|  | chr11:47353704-47353843 | GGCAGGGCTTTCTAATCTCCAG | ATGGCTTCCCTCCCTCTCTTTA |
|  | chr11:47362501-47362683 | GGTGGGTTGCGGGAAAGT | CCTGCTCCAGGGTAGGGT |
|  | chr11:47367603-47367808 | GGACTCCGCTCTTTCCATGTAT | GCATGCTAAAGAGGCTCAAGGG |
|  | chr11:47364086-47364270 | GTCCAAGCCCTAAAGCCTCAT | CGGGAGGAGACCTTCAAATACC |
|  | chr11:47356694-47356849 | GGCAGGTCCTTCACCAGTAT | TGGAGATTCCTATCAGAGGAGTGG |
|  | chr11:47354338-47354471 | CTGGTTGGAAGAATGAGGGTACA | GCTCATCATTGGCAATGGCTAC |
|  | chr11:47360112-47360264 | GGGTTCTTCACTGTGACCGT | ACTTGGATCTCACCCCAACTCT |
|  | chr11:47354993-47355187 | GCAGTGGACTGGAAAATGTGAG | CGTGCATTCAGGCACTTACCA |
|  | chr11:47355398-47355622 | ACATCAGTCCACTGGATGGGA | GCTGTGGAGCCCCTCAGA |
|  | chr11:47371276-47371494 | GCTCTCCATGTCCCCTCTCT | CTGTCGCCCCTGCCTTT |
|  | chr11:47354599-47354779 | TTGGCATCTCCACCCCTACTAT | GGTACACAGTGCAGAAAGCC |
|  | chr11:47368967-47369091 | CAGGACTCACCTCTTTTTCAGCA | TCCTGCTCCTAATCCCTTTCCA |
|  | chr11:47372910-47373105 | CCAGGCCGTACTTGTTGCT | GGTGCACGCTCCAACCA |
|  | chr11:47369336-47369554 | CCCCAGTCGAGACCCTGAA | GCAAGTCTGTGAATACTCAGAGG |
|  | chr11:47362760-47362916 | GCAGGTGTGACGTCGTCAAT | AAAATCAGAATACCAACAAGCCAGGA |
|  | chr11:47361022-47361240 | TGCTTCTTCCACCCCCTGA | CCTGCTCCCACTGTGATCT |
|  | chr11:47359086-47359302 | GCATCCACCGGTAGCTCTTC | AGAGGACTCCTGCACAGTACA |
|  | chr11:47365051-47365252 | CATCTGGATCTCCTGGCCATTC | GCCACAGCCACAGTAGCTT |
|  | chr11:47374076-47374290 | CTCGTCAACCCCCTCAAGAA | GGTCCCCATATATAGTGGGAAGGA |
|  | chr11:47364519-47364741 | CCCCTGAGGCCATCTCCT | GGGTGTCCGCAGCTTTC |
|  | chr11:47362576-47362800 | CTTGACCTCTGCAAGAGAAGGAA | TGCAGGGTCCACAAACTGAC |
|  | chr11:47360807-47361013 | CTCGGCACCACGTAGGTA | GCGCTTCCAGGCTGAGT |
|  | chr11:47369111-47369311 | GAGAAAGGGACACTAGCCAGATT | GGCGGAGCTGAAGTCAGTG |
|  | chr11:47371525-47371680 | CCTTCCCACCCCAATGCT | AAAGCCTTTGCTCACAGGGT |
|  | chr11:47367765-47367941 | CTTCTTCTCATCGCGCCTCAT | GCCTAGACTGCGGGACAC |
|  | chr11:47364228-47364422 | TCTGCCCGTCCTTCTTGAAC | GTTTGAGTGTGAAGTATCGGAGGA |
|  | chr11:47356556-47356734 | AATGGCGGGTCTTGTGACT | GGGCTGACAGAGCACACATC |
|  | chr11:47363522-47363745 | CACCTGCCCTGCACACT | CACCCTCCCGAGCTCAATG |
|  | chr11:47354431-47354639 | GGCTGAAGACGCGGAAGTA | TGCTGATCTGAATCCCTCCATAGTAG |
|  | chr11:47359930-47360153 | GGGCAGAAAAACCTGTCCTGTT | GAAGGAAGATGAGGGCGTCTAC |
|  | chr11:47369899-47370113 | AGTGTTGGGAAAAGGAGGTAGGA | CCCGTCCCTCCATGCAC |
|  | chr11:47354090-47354281 | CCTGGACCAGCGCCTAAAG | TCTCGGTACCAAGTCCTGTCA |
|  | chr11:47353531-47353745 | GTGCTTGGCCGAGGACA | GCAAGCAGGGAGTGTTGACT |
|  | chr11:47357376-47357600 | AGGTGAGCATGTTCTTCCTTTGG | GCTTGCTCAGACCCCTCTCT |
|  | chr11:47361198-47361373 | GTACCTGCGTGATAGCCTTCTG | CGTTTCTCTGAACTACATTGTGTCTTCT |
|  | chr11:47354738-47354911 | GGCTCACCATGGTCTTCTTGT | TGACCCAACTGGGTCTGTCT |
|  | chr11:47372766-47372952 | AGGGAAGGCTGATCAGGATCT | CGCGGAGGCAGTGACATC |
|  | chr11:47368797-47369011 | ACATAATGTCTGCAGAGCCCTTT | GGGATTCTGGACTTCAGCTCAC |
|  | chr11:47355144-47355335 | TGTTCTCAATGCGCACCGT | CAGGTTCAGGGTTAAGCTTTTCCT |
| MYH6 | chr14:23871800-23871999 | GTCAAAGGCACTCTGGGACA | GGTCACCAACAATCCCTACGA |
|  | chr14:23863360-23863576 | ACTTCTCCAGCGTCTCTTTGATG | TCTCTTTTATAGTGCCCCACCCT |
|  | chr14:23876272-23876474 | CGGGACAAAATCTTGGCTTTGA | GTAACATAGCCCTCCTGTCTCTGA |
|  | chr14:23855506-23855695 | CCTCCCGCCCCCATGTA | GGACTCGCTGCAGACCTC |
|  | chr14:23866312-23866535 | AGGGTCAGCCTTAGGGTAAAGT | GGACCAGGGTTTAAGGGCCTA |
|  | chr14:23858742-23858966 | TGTGGGCCATTTCACAAGTCAT | CCCTCCCACACTCACCCTT |
|  | chr14:23862073-23862296 | CAGGCCAGACACCTCCATTAG | ACACTTTGCTTATTTTCTTCCCTCCA |
|  | chr14:23867840-23868027 | GTGCCTGCCTATGGAGTCAT | CCTCTCAACGAGACTGTTGTGG |
|  | chr14:23868115-23868285 | CTTGATGTTGCGTGGCTTCTG | CTCAGTGATTCTCTCTTTGCCTCTT |
|  | chr14:23859586-23859745 | GCAGCTTCTCCACCTTAGCC | GAACCTAAGTTCCTGGTAGCTTTTCA |
|  | chr14:23853766-23853957 | AGCTTCTGCAGCTGCTTCTT | ATGTTCCTTGCCACCTCTCTC |
|  | chr14:23857066-23857245 | CCTCCTTCTGTGAGGACTCCA | ACCAGGGACAGATCTTGGACAT |
|  | chr14:23852374-23852590 | CATTGGTTCTCACAAATAGTAGGTGTGT | GGGAAAGGTGATTGCATTTGCTC |
|  | chr14:23871585-23871794 | AAGCCTCAGAGTCTCTGGGAT | GGGCTTCACTTCAGAGGAGAAAG |
|  | chr14:23857420-23857594 | CTACGTCCACCATCAAGTCCTC | GTTGCCCAGTAGAGTCACACA |
|  | chr14:23872492-23872716 | CATGCAGGAGTCGTTGGG | CTTTTTCTGGCTCTACTCTCTTTCCTT |
|  | chr14:23851138-23851353 | CTACTGCCCTGATCCAGGATG | CTCTGAAAGCCCCAGGGATT |
|  | chr14:23862565-23862788 | GCTCCAGCTTGACCTTAGACTT | CCATAGATGTCTCCAGGCTGGT |
|  | chr14:23874835-23875015 | CATATATCATCCAGGCCGCGTAG | TGGTCACTCATCCTCCTGCTTAT |
|  | chr14:23862918-23863142 | GTCAGCTCCAGGTCATCAATGT | CTTCCTCCCTGTTCTCTTCTCC |
|  | chr14:23873942-23874073 | TTCTTGCCACGGTCACCTATG | CAGTAGGAATCCTGAGTTTTGATTGGT |
|  | chr14:23856669-23856890 | CCCAGGTGAGGGAGAAGTG | TTGAGCTTTCTGGCCCTCTG |
|  | chr14:23865836-23866060 | AACTCCTCTCTGCTCCACTCA | GAGCATGGGTGACTCTGGAC |
|  | chr14:23873422-23873643 | AATCAACTGGGTGTGGCAAAAC | GGAGGTGGATGGAGGATGAAC |
|  | chr14:23855089-23855309 | GGCTCTGCACTCAGTAGGTTT | GTGCCAACGACGACCTGA |
|  | chr14:23858180-23858354 | CCTCTGTCTCCTCCTCGTACTG | GGCTTGGTTGAAGTACTACATAAGAAGA |
|  | chr14:23874423-23874618 | CAGGCTCACCTGTCAGCAT | CCTCATGCCCAGCCTTGTC |
|  | chr14:23869868-23870044 | GTAGGAGCAAGCGAGTGATTGT | GGCCAAGGCAGTGTATGAGAA |
|  | chr14:23866126-23866339 | CCTGGCTCCCCCTGTTCTAT | GCATCCACTTTACCCTAAGGCT |
|  | chr14:23863200-23863407 | CCAGTCCCTGGTTGTGAGAT | CCACCATGAAGGAAGAGTTCGG |
|  | chr14:23876175-23876315 | CTGCATCTTCTTTCCCAGACCT | CCCGATGACAAGGAAGAGTTTG |
|  | chr14:23855655-23855875 | GGCTGCGTGTCTCTGCAT | GCCCTGTGCCCTGTCTG |
|  | chr14:23851589-23851812 | GGCATGCTAATGTGGAAGTGACTA | CATCTCCAGCTCATTCACCCA |
|  | chr14:23866630-23866853 | ACGACTACGTAGCCCTAGCTT | CCTGTTGATTATTTCTCCTCTCGCT |
|  | chr14:23865458-23865680 | CTCTAGTGCATGCCTCCCTTT | GGATACTCCCCTCTGAGGCT |
|  | chr14:23867984-23868158 | AGGGAGGACTTCTGGTACAGG | ACCTGGGCAAGTCCAACAATTT |
|  | chr14:23854105-23854320 | GAAGGTGGGCGGTCACT | GGAGAAAGGGTATGAAATCAGGTAACAA |
|  | chr14:23859464-23859672 | CCGCCGCATCTTCTGGAA | CTTTGTGTCTGACCCAGGCA |
|  | chr14:23869443-23869664 | GGTGGTGAGGCCAAGGA | ACTGCTCCCACCCCTCAT |
|  | chr14:23861712-23861910 | GCCTCAGTTACCTCAGGGCTAT | AGGAAGGAAGGCTACCTGTCA |
|  | chr14:23853600-23853810 | GCCAGAGAAGAAACTTCCACATCT | CGAGCAGATCGCCCTCAA |
|  | chr14:23856931-23857110 | CCAACTCATCTCTGGCCTCTTG | AGTATGAGGAGTCGCAGTCTGA |
|  | chr14:23872827-23873051 | AAAACAGGAGATGGCAGGAATGAT | AGAGGGCCAGGATTTCACTCT |
|  | chr14:23857290-23857463 | GCCTTTGGCCTCTCACTGAA | GCACCGGCTACAGAATGAGATA |
|  | chr14:23874245-23874464 | CCCTAGGCATCAGCGTGTATG | TCTCCGACAACGCCTATCAGTA |
|  | chr14:23862764-23862961 | ATCACCAGCCTGGAGACATCTA | GAGTGCTCAGAGCTCAAGAAGG |
|  | chr14:23858556-23858771 | CCCAGGGCTGCCATCAA | GGCAGGACATGACTTGTGAAATG |
|  | chr14:23855269-23855475 | CTCCACGATGGCGATGTTCT | TGGAGAAGCAGTGGTGTCTG |
|  | chr14:23858021-23858226 | GCCCCTTCCTCTCTGAGAGT | GCATGACTGCGACCTGCT |
|  | chr14:23870002-23870212 | CGTCACCATCCAGTTGAACATC | TCACTTATCCTTTCCCTCTCAACCA |
|  | chr14:23871747-23871944 | GCTCCCGTCAGCTTGTAGAC | GCCTCCATTGATGACTCCGA |
|  | chr14:23871959-23872179 | CCTGAGACACGAAGGCGTAG | CTCCTTGGTCCTTGCTGACTT |
|  | chr14:23874668-23874881 | TGTTCACTCCAGCTGGCTCTA | GGTGCTTTTCAACCTCAAGGAG |
|  | chr14:23862393-23862606 | CACAAAGGATTCAGAGACCTACTGTA | ACAAGGTCAACAGCCTGTCC |
|  | chr14:23873761-23873985 | TGTCTCCTGTCAGGAAGGTCTT | CAGTACTTTGCCAGCATTGCAG |
| MYL2 | chr12:111358257-111358411 | GTGGTCCCTCGCTTGTAGT | ATTTATTGTTCCTGGGCTGCAGA |
|  | chr12:111353457-111353669 | TCCTGCTCCTCATGGATGTGA | AGTCCTTGGCCCAATCTCTTG |
|  | chr12:111350955-111351167 | AGAAAGGAAAGCAGGTGTTGGT | GCCACCCCCAGTACATGTAAG |
|  | chr12:111348830-111349040 | GCAGGGACCACTCTGCAAA | CCCACCCTCCGTCTCAGT |
|  | chr12:111351933-111352157 | CCCCCGAAGAAACATAGACACATA | GCAGTGTCATAATGGTTCTTTCTCC |
|  | chr12:111356858-111357081 | CTCGTGGGTGGGATTTCCAT | GATGAACACACCCAGAGTAGGG |
|  | chr12:111350785-111350999 | GCTGGTTCTCTGTCAGTGTGG | CCCCCTACACACACACAACTG |
| MYL3 | chr3:46902116-46902328 | CCAGTCTCCCCGGTACTAACA | CCTCTCCTGGCAGAGTTCAAG |
|  | chr3:46899660-46899881 | CTGCAGCTGGTGGAGTGT | CTATGAAGGTGGGCTCAGCAG |
|  | chr3:46900901-46901094 | ACAGAGTGGTTTCTCCCAGGAT | CTCCAGCACATTTCCAAGAACAAG |
|  | chr3:46902359-46902583 | CCTACCCCACTCCCCACA | GACAGGCTGAGACAGTTGACATT |
|  | chr3:46904706-46904929 | CTCACTTGCCCTGCTCCTATT | GCTTTCTGCATTCTTCTCTCCACAT |
|  | chr3:46899717-46899901 | CTACCTGGGCACGAGGTTTA | GACTCCAATGGCTGCATCAAC |
|  | chr3:46901050-46901256 | GTCCTCATAGGTGCCTGTGTC | GGTGTAGACTGTTGTAGAACTGTGT |
|  | chr3:46902286-46902469 | GGTCGAACAGCATGAAGGCTT | CACACCTGAGCAGATTGAAGGT |
| MYOZ2 | chr4:120085361-120085573 | CAAAGGATATTCTGGACCACTGAAGG | TGTCACCCAGGACCATTGAATT |
|  | chr4:120072057-120072234 | TCAGCATCCCCAGAGACATCA | GCATTCATTTGGATACTACCTGTTCAAG |
|  | chr4:120079173-120079370 | ACAGCACAGTATTGCTATGCAGAA | GCCTTGAGTCATATCATTAGGTACACA |
|  | chr4:120107109-120107255 | AATTTCCCACAGGGTTGCCA | AAGGACCGTCTGCCAGAAAG |
|  | chr4:120057610-120057819 | AGACACTCCAAATGAGTTCTTCACATT | CGTCAGGATGGAGCCACATTC |
|  | chr4:120071983-120072107 | ATGCTTACCTTGGGATTTTTACTCATATGAA | GGTTACTGAGATGGGATAATTCTTCCAA |
|  | chr4:120107211-120107434 | CAGGTTTATGTCCTTTGTCAATCCC | AGTGGCTTTGCCAGTAGTTAAAATAGT |
|  | chr4:120085211-120085414 | ACACTTGCAGACTGCTGTTTTCT | GTGGTGTTGAATTTTTCAGGAGGAATTT |
|  | chr4:120106935-120107154 | TCTTTAAAGTAGAAATGCTTGCCTCAGA | TGCTTTTTCAAAACCTCCAAATGGTG |
|  | chr4:120079004-120079221 | CATTCAAGTGCGCAACCATCT | CAAGTTACTTCCATCCACTTTCCCA |
| NEXN | chr1:78383598-78383740 | GTCTAATTTTTGGACATGTGCTCACATT | CCCCTCAAGCTTCTTACCTGTTAATTT |
|  | chr1:78401671-78401877 | CAAGAAGGAGAGCAATTGACCTTGA | ACATGTGTTTTGCTATTTACTTCCCAGA |
|  | chr1:78383253-78383448 | TCTGCTTTCTTCATCTAAACCTGTCC | AATAACCTCCTGCTTTCTCCTGTTC |
|  | chr1:78392430-78392578 | AGAATCACTTTCTCCCGGAAAATTGA | CCATTTGCCGCCTTGCT |
|  | chr1:78381733-78381887 | TTTTCAGGTGCAAATATATACAGAGCTTCA | CCTTTCAGCAATTCTACAGTCAACTCTA |
|  | chr1:78399101-78399303 | ACGGAAGCATAAGCTAGAAATGGAG | CCTGTACCATGTAAGCACCTCATAATA |
|  | chr1:78408228-78408435 | CTCCATGGTTCAAGAAGCCTCTTA | CCTCCATCTTCTGGGAAAGTTTCTG |
|  | chr1:78383809-78384005 | GGAACTGTGAAGGGTAGATTTGCT | GCACAGTTAGTTTCAGAAACAAATACGA |
|  | chr1:78407834-78408008 | GAACAAAAGTTACTACGCATGCAGT | ACATTCCAGTGACTTGAAAGAAAACCA |
|  | chr1:78392111-78392280 | TCACTACTTATAACTGTGGTACCTGTCAA | CACTTTGCTTCCTTGAGAGATGGT |
|  | chr1:78395000-78395176 | GGTAAATGAAGATGAGGAAAACCAAGACA | GCTTCAGCAAACGCCTTCTT |
|  | chr1:78390875-78391069 | TGAGGACATAAACAATACGGGAACTG | AAGTATGCTCTTGCTTTGCAAAACAT |
|  | chr1:78383656-78383860 | GCTTGCTTCTGATGATGAGGAAGAT | GTTCCTCTTGTCTTTGTTTCTCCATTTC |
|  | chr1:78401507-78401723 | GGAAGAGGAAGAAAATGAAACCTTTGG | AAAATTTTCAGCTTCTCGCTCTTTAATT |
|  | chr1:78398966-78399153 | GGTAGTAGATGATGACTCCCCAGA | CCTGTCTCAGTTGTTCAAATTCTTGTTT |
|  | chr1:78408079-78408278 | ATTGTTACTAAATCGCTGCCCTGA | GCTCACTGTCTACAACTGATGTGTTTT |
|  | chr1:78408385-78408603 | GGGAGAAACTTACTGCCTTTACTTAC | GGGAGCTAGTAGTCATCAGCTAAAAA |
|  | chr1:78407676-78407883 | GCAATTGTTAATCTTGGCCCACT | GCTGCATCAATTTCCCTTTGTTCAA |
|  | chr1:78391994-78392216 | ACATTGATGACAGTGTAATGAAATTCACCTA | TGATCCTTTCTTTCTCTTCACGTTCTTT |
|  | chr1:78394891-78395059 | GGCATTGAAACTCCTGTGTGATAG | CCAGGGCGGTACCCTTTAA |
|  | chr1:78390711-78390928 | AAAGGATATATTTGGCTAATTTGGAACAAC | AAATGTCTGTTTACCTCTGATGCTGATT |
|  | chr1:78383087-78383305 | AGAAAGTATCTAATCAGGTTGTGATGGT | TGCCAAGTTTTGGTACATAGGTTTTTG |
|  | chr1:78383407-78383620 | ATTAGAGAGAGAGAATGGAACAGGAGAAA | GAGCACATGTCCAAAAATTAGACTAAATC |
|  | chr1:78392224-78392353 | GGAAGATAAAAGAATAAGATATGAAGAACAACG | CTGCTAACTTAAACTGCCTTGATTTGT |
| PLN | chr6:118880041-118880255 | CTCTCGACCACTTAAAACTTCAGACT | TGTAGCAGAACTTCAGAGAAGCATC |
|  | chr6:118880336-118880525 | CACTTCCTGAGTAGAAGAGTTTCTTTGT | TGGGATAGAAATTTGTGAGCCATGT |
|  | chr6:118880838-118880996 | TTTAAAGTTGATGAGAATCAAGTATGGAAAAG | CACCAGTTCTCATCTGTTGATCATATGT |
|  | chr6:118880554-118880742 | TTTAAAACTGCACTGCCAACAAGTT | GTGAGGAGTCAGTGGACTATTTTGAATA |
|  | chr6:118880991-118881215 | CTGGTGGTTAATATGTGACAGTGAGA | GGAAATCTTTTATTTTCCTTGCCTGCAT |
|  | chr6:118880490-118880684 | TGATTTCCTCAACATGGCTCACA | ACTTGGTGAAGACCTGAAAAATACTT |
|  | chr6:118880729-118880887 | CACTGACTCCTCACATCTGTTATCTT | TGTAAGAGTATGGCCTTACTTTTCCAT |
|  | chr6:118881190-118881339 | GCAGGCAAGGAAAATAAAAGATTTCCA | CTGTAGATGTAATAGATGGGCCAACA |
|  | chr6:118880916-118881088 | CAAAGAATCACAGAATTCTAGTACATGTAGGT | GCTCAGATAATTCACTACAGTGCCTTAA |
|  | chr6:118880206-118880425 | GTCTCTTGCTGATCTGTATCATCGT | CAAGATCCAACAGATGAATACATATGGT |
| PRKAG2 | chr7:151262780-151263004 | TGCCTGTCAGCGCCAAA | GGTCTAGAACGTTTGCGTTTATCTTC |
|  | chr7:151254202-151254426 | CACTTGCAGCCAGTGTTCAT | TCTGGATCATCCTCCTAAGGCA |
|  | chr7:151273365-151273564 | GTTGAATTCCAAACCGGCATCTAT | GTTTTGTTTCTCCTCTTTTTCTTCAGGT |
|  | chr7:151478201-151478407 | CCAGCACCCACCTGAAACAATA | CGGCTCTCCCAAAACCGT |
|  | chr7:151265785-151265972 | CGCTAAAAACTACTTACAAAAAGCTGGA | TTTTCTCGAAAGCCTCCTAACAACTT |
|  | chr7:151483511-151483706 | GCACCTCAAGTGAGCTGTGAG | TGACACCCACGTAGAGTTCCAT |
|  | chr7:151329144-151329268 | GGCGCACTCACCTTCGT | GCGTCGCAGCTCATGCT |
|  | chr7:151372494-151372644 | GCCCAGACTTACGGCTTTGG | CGAGCCTGAACGGTTAGAGAA |
|  | chr7:151269655-151269799 | CAGAGAGAAAAGGATCTGCAAAATGAA | GCAGGTACAGATTTATGAATTAGAGGAACA |
|  | chr7:151271840-151272040 | GAAAATCACCATCAGCACACCAT | TCCAGGAATGCTAACAATTACAGATTTCA |
|  | chr7:151261086-151261219 | GCTTCCAGAGGCATCATTCACTA | AGTGCAATAAGCTGGAAATACTGGAG |
|  | chr7:151292346-151292546 | CTGGCATTGCTGGTTTTAAATTACTGT | TTTTAGCAGTAGAAGACTCAGAAAGTGG |
|  | chr7:151267150-151267374 | CCCTGTTTGGAATGAAGAACATGTTTA | TTGTGTTATAACTGCAAACAGCAAATGT |
|  | chr7:151573573-151573739 | ACCGAGTGCTGGGACTCA | AGGAGTTTCGCAGAATCAACTTCT |
|  | chr7:151478370-151478547 | CCTGGTAGGAGAACGGGAAC | GACTGCATCCATTCGTCTGTTTTTC |
|  | chr7:151372603-151372748 | GAAGACGAGGCATAGATGCGA | ACCGTCCTTGGTGTTTCTTCTG |
|  | chr7:151257521-151257742 | GAGAAATGATGGTTTAAATGCTGCACT | AAATGTTAATTTTCACGTGTCCTGCTTT |
|  | chr7:151271965-151272129 | GGTGCCACTTACCATAGGTGATTTAT | GCTCTTCTAGCATATATTCTAGGCTCAC |
|  | chr7:151261169-151261390 | CTCTTACTATTCTGTCCACGATGGT | GCTCTCTTAAGCTCACTAATTTTGGTCAT |
|  | chr7:151265647-151265837 | TCAGAGAGTAGTAAGTTGAGAAACTGAA | ACCCACAAAAGAATCCTCAAGTTCC |
|  | chr7:151269743-151269881 | TGCTTACCCCTCCATGTTTCAATTTTA | ATCCAAGCTATGTTTTGTTTCATTTTCCA |
|  | chr7:151372326-151372533 | TTGCTATTGTGCCCTCACCAT | ACCGACACACTATGCTCCCT |
| TCAP | chr17:37821535-37821756 | CCGAAAATAGCCCCTGGAGA | AGCAGAGGCAGGGCTCTA |
|  | chr17:37822097-37822301 | AGTACCAGCTGCCCTACCA | TTGCTGACAGGCACCACA |
|  | chr17:37821913-37822137 | CCTGTGCCCAGAGAGCAA | GATGGGCAGCGGCAGTA |
|  | chr17:37822254-37822470 | GGTGGCTGAGATCACAAAGCA | CTCTGGGCAAACTACAAAGCAG |
| TNNC1 | chr3:52487929-52488096 | GCTACTAACCCCGCACTCTCA | GGGCAGGGCTATTTAAGTCAAG |
|  | chr3:52485186-52485399 | GGACATGGCCAGGCTCA | GGTGGGTGGGCTGATCTC |
|  | chr3:52486115-52486310 | GGCTCACCGTCCTCGTC | TGCTGAGCCCTGACTACTGT |
|  | chr3:52485486-52485698 | GCCTGTAGCCTGCAGCATTAT | GCAGTCTCAGATTCCAGGCCTA |
|  | chr3:52485705-52485928 | GTACAGCTCGGCTTGAGTGT | GCTGCCTGCAGTACCTACC |
|  | chr3:52485304-52485528 | TTCATGAACTCCAGGAACTCTGTG | CATCGACCTGGATGAGCTGAAG |
|  | chr3:52486400-52486622 | CCAAGCCTCTGGTCTCTGG | TCCAGCTCCAGCCTTCTCA |
| TNNI3 | chr19:55666066-55666243 | CTCACCCTACCCCGAAGGTA | CTGTTTTTGGTTCCCCCAACA |
|  | chr19:55665365-55665577 | GCATCCTCTTTCCTGGCCTTA | CAGATTGCAGATCTGACTCAGAAGAT |
|  | chr19:55668835-55669059 | CAAGAGTCCCTACGCCTACCT | GGTCCCTGGGACCCTGAA |
|  | chr19:55667877-55668087 | CCACTTCCGCCCACCTA | TGTCTTGAGGTCCCCTCCA |
|  | chr19:55665527-55665680 | CTTAAACTTGCCTCGAAGGTCAAAG | CGGTACTGGAAGACGAGATAAGGA |
|  | chr19:55663128-55663352 | GAGTCACTTTCAGCTCAGAGAGAA | ACAGGGATTCTTGAGAGACTGGA |
|  | chr19:55667535-55667758 | GGACTAGAAACCTCGCATCCTT | GCCTGGTCTTTATCCTGAAGCC |
|  | chr19:55668355-55668568 | CCACTCCCAGGGTCTTGGAT | GGGTGCGGTACGGTAAGG |
| TNNT2 | chr1:201333375-201333566 | GGAGCTGGGAGCATGGG | CTGTACCTGCGATGTCACCTT |
|  | chr1:201331134-201331357 | CCACTTTTCCGCTCTGTCTGG | AGGAAGAGACTGACGTGATCCT |
|  | chr1:201328227-201328448 | ATTTCCAAACAGGAGCTGCCT | CAGCCGCATGGTGACCTA |
|  | chr1:201338401-201338597 | CCCTTGCCTTGGTCCTGTG | CACCCCAGTGTTCCATGCT |
|  | chr1:201342223-201342446 | CAAAACACACACAGCTACTTCTACC | GAAGGCAGGCTTCCCTTTGTA |
|  | chr1:201341062-201341267 | CAGGGACAGATGAGCTGCTTT | CGTAAACGTGTGTACTCATTTGGATCA |
|  | chr1:201330333-201330550 | CAGTATTACCGGACCCAGTGAA | CCAGGAGGGCCCTTTCTTACT |
|  | chr1:201334680-201334899 | TGAGGGCCCTTGGGACTAT | TGGAAGATTCTCTAGGAAGGATCAGG |
|  | chr1:201336816-201337038 | AAAGAGCACTGTGGGCATTCT | CATCTTGGCTAGGGCTTATCTGTG |
|  | chr1:201335872-201336090 | CCGTGTCCACTGCACCATAC | GAAATCCACAGGGATCTAGCTCA |
|  | chr1:201337210-201337431 | GGGTTTCTTACTGCCTCAGGAAT | CCATTCTCTGCTCTGGGTTCTG |
|  | chr1:201332363-201332583 | ACACCTCATTCCTCAGGGCTA | TTCCAATCCTTTCCCCTAATTTGCT |
|  | chr1:201328666-201328884 | CCCTCCAAGGAGGAATGGGATA | GCACTCAGCCCCCTTCTC |
|  | chr1:201341198-201341416 | GAGAAGAGAGAAGAGGTGGGTCA | CTCCACTAGGCAACAAGGGAAAA |
|  | chr1:201334271-201334490 | ACAGACTGGGCCATCAGAGAA | TCTCCTCTGGACTCTTTGGAGTG |
|  | chr1:201338809-201339033 | GAGGAAACGACTGACCCACTC | TGCTCCCAGACTAACCTGTCT |
|  | chr1:201330986-201331191 | CCCCTCCCAGAGCAGATG | GGGCAATCTGGCCAGTTTACT |
| TPM1 | chr15:63353001-63353210 | CCCATGCCCTTCTGTTACACA | CAGAAGGTCATGCTGTTTAGTCACT |
|  | chr15:63340605-63340827 | GCTGCGACTTCCGGACT | TGCAGGCTCCGGATCTTC |
|  | chr15:63354687-63354911 | GCTTCATTTTCATCCTCTAGTTTTCCC | AAAATTGTTTTGCCAGGTTGGTATTCA |
|  | chr15:63353847-63354070 | CCTTTTTCTCTCCTCCTTCCTTTGG | ACTGTAAGTGTTGCTTTCTGGCA |
|  | chr15:63363181-63363402 | ATCATCTCATCCTGTGTTTGTGATTGAT | CGAAAGAATGTGGTCGCAGCTA |
|  | chr15:63349139-63349358 | CTCACTTTCTCCCCAACTCTGA | GGAAAGGCAGCTGCAAAAGATG |
|  | chr15:63357989-63358213 | GTCTGTGTTTCAAGTGCTCTCATCT | GCAGGATGCTAAGAGAGAGAACCA |
|  | chr15:63336057-63336269 | GCGCACGAATGGCTAACTCTT | GTGCCCTTGAGTTTCTTTTGCAG |
|  | chr15:63334870-63335065 | AAAAGTATTGGCTGTCTTGAGGAAT | GCTTCAGCATCTGCATCTTCTTCT |
|  | chr15:63335836-63336006 | CCCGTGTGTTGTGTGTGTCTAA | CAGGAGGCTGTCCTCCG |
|  | chr15:63361997-63362216 | GCCTCTCACCAAGTCTGCTAA | GGTAGGACAGTCTTAGATTTTCTGCAC |
|  | chr15:63354336-63354545 | CTGGCGAGTTCTTTGCATGAG | CCCTGACCGGTTCCATGAAAA |
|  | chr15:63356192-63356415 | CCTCACTCACCCTCCATTTCTT | GGAAGGCATGGTGGTGAGTTTA |
|  | chr15:63335024-63335200 | CCACCATGGACGCCATCA | GTGCCAGGCTCGAGTCC |
|  | chr15:63340781-63340928 | GGGAGTAGCTCGCTGGA | GGGTGATGGGTGTATCCCTTA |
|  | chr15:63353322-63353541 | GCGTGTATCACTGCATGCCTTA | AGGAGGCATTGGAAGCTGAAAG |
|  | chr15:63336226-63336449 | CTGGAAGATGAGCTGGTGTCA | GAAAAGGTAAACTTCCTTCATTGCTCTC |
|  | chr15:63335970-63336154 | GCTGGAGGAGCTGCACAAG | GTGGGTGCACAGAACCAGAA |
|  | chr15:63351705-63351921 | TGCATTTGGGAAGTTCAGCTCTA | CACTGCTGGGTGTCCACAA |
| VCL | chr10:75854137-75854266 | CAAAGCAGCTGTACACCTTGAG | TGCACTGAGACCAATTTAATGAGGTTAT |
|  | chr10:75868745-75868963 | CTCCTCCCAAACCACCTCTG | CCTTTCCGAGAAGACATCCTTTCT |
|  | chr10:75832454-75832677 | ACTCACCCTGCACAATTTCTTCTT | GAACCTCAGAGTTTAGAACATGCTAACA |
|  | chr10:75834535-75834696 | AAACCAAGGCATAGAGGAAGCTTT | GGGATTTTGCTTATTTCTCCACTAAGA |
|  | chr10:75848913-75849137 | TTTGAGGGTGTACAATGACAGCAT | CCCACCCCTCCTGAAAATCAC |
|  | chr10:75877787-75877993 | GAGATGCTGGTTCACAATGCC | TGGGACTCAGATCATTTTCCTTCTTTTT |
|  | chr10:75871746-75871920 | CCCCTGACATGGAAGACGATT | ATGGCCGGATGCATATGGAG |
|  | chr10:75860531-75860754 | GAGATCCATCAAGGCCAGGAT | CAAGCTTTCCTGAATGGTTTTCAAAGT |
|  | chr10:75830396-75830614 | GCCGGTGTGTTAACCTGTGTT | TCTTCAAGAGTAGGCTTTTTGTCTCAG |
|  | chr10:75864812-75865029 | TGGTGGACGAAGCCATTGAT | TTTCACAGCCTCACGGAACTT |
|  | chr10:75855450-75855674 | ATCGTCTGGCTAATGTTATGATGGG | CCCTCGTCTTTCAATAATAGGAAGCATA |
|  | chr10:75874551-75874719 | AGGTATGTGAGCGAATCCCAAC | TTAAATTACTGCAAAACGGCACAGAA |
|  | chr10:75874036-75874245 | TCATTCAGTGTGCCAAGGACATC | CTCCTGCTTTTCTCCCTCTCATAC |
|  | chr10:75863536-75863750 | GCAAGGGTGCTCTGGTGTTTA | GCTCCAATTTCTCAGAACTCATTCTTCA |
|  | chr10:75842969-75843181 | CACTGCTTGGCTGTTAGCTATC | TCACCAACTTTTCCAGCTTCATCT |
|  | chr10:75866948-75867156 | CTGACCCACCCAGCTGAAA | CAACACCTTCCTGCTCAATTCTCT |
|  | chr10:75849881-75850097 | AAGAAAGAGATGACATTCTACGTTCCC | GGAAGAGCATCATAAGTGCATTTAACCT |
|  | chr10:75757877-75758006 | CGCTGCACAGTCTGTCTCTTC | TCCAGGATGCTCTCGATCGT |
|  | chr10:75802664-75802880 | TGTGAAGGCATATTATGTGAGTAATCCT | AAATCTGATCCTCAGTGGTTTGAACA |
|  | chr10:75856913-75857136 | GCTTTGAGGATGTATCTGGACATTTTCA | CCTATAGCACAGGCTGACAGAAA |
|  | chr10:75877629-75877829 | GTGATCTGGGAAAACCCCTAGT | TCACAGACTGCATGAGGTTCTG |
|  | chr10:75830671-75830895 | TGTGTTACCGTGTTTGCTAGTTGT | GGCTCCAACATGGACAATCCTTA |
|  | chr10:75871570-75871788 | GGCTTTGGTTACTAAACCAGCTGAA | ATTAACAGCAGCTCAGGTTCGT |
|  | chr10:75860704-75860880 | AGGTATTTGATGAGAGGGCAGCTA | GTATGAGGAATGAGCAAAACCCAAC |
|  | chr10:75864645-75864855 | TGTGGTGTTTACCTGGAAGCTTT | TGAAGCATCCAACAGAGATTTGGT |
|  | chr10:75864988-75865136 | GTGGAGAATTCCGAGGATCCC | AGACATAGTGCCATGCATTGCTTA |
|  | chr10:75855337-75855498 | CCACATCGCACCACAGAATG | GCGAGAAGATCTTGCCGATAAGG |
|  | chr10:75874378-75874595 | GGAGAAATGGATTGTACTGACCCT | GGATTTTGAGCTGGGTGCTTATG |
|  | chr10:75873862-75874081 | TCTTCTCTGTGCTTCTCCTTCCTT | GTCACCTCATCTGAGGCCTTG |
|  | chr10:75843137-75843307 | GCAGGCCATCAGACAGATCTT | CCCACTACTGCTCTGTAAGGACA |
|  | chr10:75849724-75849932 | GTAGAAAGGAGTGTGTGAGTAGATCAC | GAAGTCAGAGCAGATATTTCCCCAA |
|  | chr10:75802833-75802983 | TCTTGTAGGTTGGAAAAGAGACTGTTC | AAATCAGCTACTTTTTCTTCCTAACAAGAGT |
|  | chr10:75854003-75854179 | CAGGATGGCTGTCTTTTTCTCTGTA | CTGTGCTTGCTCAATCTTGCC |
|  | chr10:75842137-75842361 | TCTTCTCTGTCTTACGTTCTATCATGGT | ATTTGCTTGGTTTTTAGCTTCTCGTT |
|  | chr10:75868565-75868785 | CTTCTGTTGAGATTGAAGCAGGT | TGGAGGGACCTCACCTTCAG |
|  | chr10:75757962-75758139 | CGCGATGCCAGTGTTTCATAC | GCTCACCCGGACGAGGTT |
|  | chr10:75834405-75834562 | AGCCCAAAACATCTAAAGTGTAGAACA | TTTTAAAGCTTCCTCTATGCCTTGGT |

| **Supplementary Table 2 Variant Caller Parameter Settings.** |
| --- |
| **SNP Caller Parameter Settings:** |
| hp-pen-slope=0.5 |
| snps-min-base-qv=18 |
| hom-min-nonref-base-qv=18 |
| het-min-start-pos=2 |
| call-stringency="medium" |
| relative-variant-strand-bias=0.95 |
| snps-min-filteredreads-rawreads-ratio=0.15 |
| het-skip-high-coverage=0 |
| het-min-nonref-base-qv=18 |
| het-lca-both-strands=0 |
| het-max-coverage-bayesian=60 |
| het-min-coverage=2 |
| reads-min-mapping-qv=4 |
| het-min-validreads-totalreads-ratio=0.65 |
| het-min-lca-base-qv=18 |
| hom-min-nonref-start-pos=0 |
| het-min-lca-start-pos=0 |
| mini-strandcount-fpr-strand-bias=10 |
| snps-both-strands=1 |
| het-min-allele-ratio=0.2 |
| snps-min-nonref-base-qv=18 |
| het-min-allele-ratio-hotspot=0.2 |
| reads-min-alignlength-readlength-ratio=0.2 |
| hom-min-coverage=1 |
| reads-with-indel-exclude=0 |
| hom-min-nonref-allele-count=3 |
| hp-jump-pos=6 |
| hp-penalty1=4.0 |
| hp-jump-amount=15 |
| **Indel Caller Parameter Settings:** |
| min allele frequency=0.2 |
| min indel count for genotyping=5 |
| hp max single peak std 23=18 |
| hp max length=9 |
| info vcf=0 |
| hp min cov each strand=3 |
| min mapping quality score=4 |
| hp stb max two peaks relative bias=0.8 |
| hp max single peak std increment=5 |
| hp stb fpe min coverage=30 |
| hp stb max avg peak srand distance=40 |
| downsample to coverage=2000 |
| hp low stringency=0 |
| fpe max peak deviation=31 |
| **Long Indel Assembly Parameter Settings:** |
| kmer len=19 |
| min var count=5 |
| max hp length=8 |
| relative strand bias=0.80 |
| min var freq=0.15 |
| min indel size=4 |
| short suffix match=5 |
| **Filter-Indels Parameter Settings:** |
| bay-score-minlen=11 |
| min-bayesian-score=2.5 |
| variant-strand-bias=0.90 |
| min-var-freq=0.199 |

| **Supplementary Table 3 Patient Characteristics** | | | | | | |
| --- | --- | --- | --- | --- | --- | --- |
| **Nr.** | **Gender** | **Age, y** | **Disease** | **Family History** | **MWT, mm** | **LVEF, %** |
| 1 | Female | 36 | HNCM | Negative | 14 | 40 |
| 2 | Male | 39 | HNCM | Negative | 14 | 51 |
| 3 | Male | 50 | HNCM | Negative | 14 | 65 |
| 4 | Female | 86 | HNCM | Negative | 14 | 66 |
| 5 | Male | 39 | HNCM | Negative | 14 | 54 |
| 6 | Male | 73 | HNCM | Negative | 14 | 47 |
| 7 | Male | 55 | HNCM | Negative | 15 | 42 |
| 8 | Male | 25 | HNCM | Negative | 18 | 65 |
| 9 | Female | 57 | HNCM | Negative | 14 | 66 |
| 10 | Male | 71 | HNCM | Negative | 32 | 54 |
| 11 | Female | 45 | HNCM | Negative | 14 | 70 |
| 12 | Male | 59 | HOCM | Negative | 17 | 71 |
| 13 | Female | 59 | HNCM | Positive | 24 | 70 |
| 14 | Male | 47 | HNCM | Negative | 14 | 51 |
| 15 | Male | 48 | HNCM | Negative | 15 | 45 |
| 16 | Male | 38 | HNCM | Negative | 22 | 53 |
| 17 | Male | 46 | HNCM | Negative | 19 | 63 |
| 18 | Male | 35 | HNCM | Negative | 15 | 23 |
| 19 | Male | 20 | HNCM | Negative | 14 | 49 |
| 20 | Male | 53 | HNCM | Negative | 15 | 63 |
| 21 | Female | 62 | HNCM | Negative | 14 | 35 |
| 22 | Female | 63 | HNCM | Negative | 15 | 30 |
| 23 | Female | 52 | HNCM | Negative | 16 | 69 |
| 24 | Male | 54 | HNCM | Positive | 14 | 85 |
| 25 | Female | 52 | HNCM | Positive | 15 | 86 |
| 26 | Male | 65 | HNCM | Negative | 18 | 72 |
| 27 | Male | 57 | HNCM | Negative | 15 | 38 |
| 28 | Male | 50 | HNCM | Negative | 14 | 62 |
| 29 | Male | 30 | HNCM | Negative | 14 | 58 |
| 30 | Male | 70 | HNCM | Negative | 17 | 69 |
| 31 | Male | 55 | HNCM | Negative | 14 | 38 |
| 32 | Male | 28 | HNCM | Negative | 24 | 71 |
| 33 | Male | 70 | HNCM | Negative | 14 | 30 |
| 34 | Male | 45 | HNCM | Negative | 14 | 52 |
| 35 | Female | 55 | HNCM | Negative | 15 | 72 |
| 36 | Male | 49 | HNCM | Negative | 14 | 57 |
| 37 | Male | 41 | HNCM | Negative | 15 | 38 |
| 38 | Male | 41 | HNCM | Negative | 14 | 60 |
| 39 | Female | 63 | HNCM | Negative | 14 | 54 |
| 40 | Male | 37 | HNCM | Negative | 14 | 18 |
| 41 | Male | 56 | HNCM | Negative | 15 | 53 |
| 42 | Male | 67 | HNCM | Negative | 15 | 65 |
| 43 | Male | 55 | HNCM | Negative | 14 | 55 |
| 44 | Male | 42 | HNCM | Negative | 28 | 58 |
| 45 | Male | 41 | HNCM | Negative | 15 | 60 |
| 46 | Male | 49 | HNCM | Negative | 14 | 67 |
| 47 | Male | 71 | HNCM | Negative | 14 | 71 |
| 48 | Male | 32 | HNCM | Negative | 15 | 48 |
| 49 | Male | 25 | HNCM | Negative | 18 | 61 |
| 50 | Male | 60 | HNCM | Negative | 14 | 73 |
| 51 | Male | 65 | HOCM | Negative | 23 | 67 |
| 52 | Male | 51 | HNCM | Negative | 31 | 62 |
| 53 | Male | 58 | HNCM | Negative | 15 | 60 |
| 54 | Female | 33 | HNCM | Negative | 14 | 40 |
| 55 | Male | 34 | HOCM | Negative | 16 | 61 |
| 56 | Male | 38 | HOCM | Positive | 19 | 60 |
| 57 | Female | 65 | HOCM | Positive | 20 | 53 |
| 58 | Male | 35 | HOCM | Positive | 25 | 49 |
| 59 | Male | 1 | HNCM | Negative | 14 | 60 |
| 60 | Male | 23 | HOCM | Negative | 32 | 45 |
| 61 | Male | 40 | HNCM | Positive | 32 | 30 |
| 62 | Male | 27 | HNCM | Positive | 25 | 50 |
| 63 | Male | 48 | HNCM | Negative | 17 | 67 |
| 64 | Female | 15 | HNCM | Negative | 18 | 68 |
| 65 | Male | 40 | HNCM | Positive | 31 | 29 |
| 66 | Male | 28 | HNCM | Positive | 25 | 51 |
| 67 | Male | 8 | HOCM | Positive | 26 | 70 |
| 68 | Male | 15 | HNCM | Positive | 46 | 32 |
| 69 | Male | 28 | HOCM | Negative | 16 | 59 |
| 70 | Female | 31 | HNCM | Positive | 18 | 60 |
| 71 | Female | 37 | HOCM | Negative | 20 | 63 |
| 72 | Female | 61 | HNCM | Negative | 23 | 75 |
| 73 | Male | 32 | HNCM | Negative | 18 | 48 |
| 74 | Male | 56 | HOCM | Negative | 16 | 66 |
| 75 | Female | 18 | HOCM | Positive | 41 | 53 |
| 76 | Female | 59 | HNCM | Negative | 13 | 60 |
| 77 | Male | 51 | HOCM | Negative | 20 | 71 |
| 78 | Female | 48 | HNCM | Negative | 22 | 62 |
| 79 | Male | 12 | HOCM | Negative | 35 | 79 |
| 80 | Male | 57 | HNCM | Negative | 19 | 80 |
| 81 | Male | 34 | HNCM | Positive | 17 | 43 |
| 82 | Male | 70 | HNCM | Negative | 15 | 68 |
| 83 | Female | 41 | HNCM | Positive | 26 | 76 |
| 84 | Male | 45 | HNCM | Negative | 15 | 31 |
| 85 | Female | 49 | HNCM | Negative | 23 | 66 |
| 86 | Male | 32 | HOCM | Negative | 27 | 73 |
| 87 | Female | 51 | HNCM | Negative | 16 | 47 |
| 88 | Male | 74 | HNCM | Negative | 19 | 63 |
| 89 | Female | 47 | HNCM | Positive | 21 | 56 |
| 90 | Male | 39 | HOCM | Negative | 16 | 65 |
| 91 | Female | 35 | HNCM | Negative | 20 | 66 |
| 92 | Male | 78 | HNCM | Negative | 21 | 50 |
| 93 | Female | 84 | HOCM | Negative | 17 | 63 |
| 94 | Male | 56 | HNCM | Negative | 15 | 66 |
| 95 | Male | 24 | HOCM | Negative | 23 | 56 |
| 96 | Male | 35 | HNCM | Negative | 15 | 68 |
| 97 | Male | 54 | HNCM | Negative | 23 | 60 |
| 98 | Female | 63 | HOCM | Negative | 17 | 64 |
| 99 | Female | 41 | HNCM | Negative | 14 | 39 |
| 100 | Male | 64 | HNCM | Negative | 15 | 64 |
| 101 | Male | 70 | HNCM | Negative | 18 | 54 |
| 102 | Male | 43 | HNCM | Negative | 16 | 41 |
| 103 | Male | 41 | HNCM | Negative | 23 | 70 |
| 104 | Male | 53 | HOCM | Negative | 18 | 69 |
| 105 | Male | 60 | HNCM | Negative | 21 | 72 |
| 106 | Female | 72 | HOCM | Negative | 20 | 61 |
| 107 | Female | 29 | HNCM | Negative | 17 | 70 |
| 108 | Male | 46 | HOCM | Negative | 21 | 65 |
| 109 | Male | 46 | HOCM | Negative | 27 | 60 |
| 110 | Female | 83 | HOCM | Negative | 15 | 64 |
| 111 | Female | 49 | HOCM | Negative | 25 | 69 |
| 112 | Female | 58 | HNCM | Negative | 20 | 74 |
| 113 | Male | 44 | HNCM | Positive | 15 | 65 |
| 114 | Male | 35 | HNCM | Negative | 21 | 73 |
| 115 | Male | 32 | HOCM | Positive | 17 | 81 |
| 116 | Male | 21 | HNCM | Positive | 16 | 72 |
| 117 | Female | 53 | HOCM | Positive | 18 | 80 |
| 118 | Female | 34 | HNCM | Positive | 15 | 75 |
| 119 | Male | 31 | HOCM | Positive | 21 | 85 |
| 120 | Male | 42 | HOCM | Negative | 16 | 80 |
| HNCM, hypertrophic nonobstructive cardiomyopathy; HOCM, hypertrophic obstructive cardiomyopathy; MWT, maximal wall thickness; LVEF, left ventricular ejection fraction. | | | | | | |

**Definition of P value of Variant Caller in Torrent Suit v3.6.2**

Two methods based on statistical modeling are implemented for base-space SNP prediction: Bayesian and frequentist. The Bayesian method is currently only implemented for germ-line SNP detection and is most useful when coverage is low to medium (10-50). The frequentist approach is used for positions with higher coverage and can be used to detect low frequency variants – for example, it is used by default for positions with filtered coverage greater than 60x.

**Bayesian Method**

When coverage is below a threshold (for example, 60x), we use the Bayesian method. From the set of reads covering the position, we have the number of reads supporting each of the four alleles. We use the most common allele, mca, and second most common allele, sca. The Bayesian algorithm checks three hypotheses: No SNP (ref), homozygous SNP (if mca is not a reference allele), and heterozygous SNP (mca/sca), by calculating P(ref|R), P(Het|R), and P(Hom|R), where R is the set of the reads, and each of the posterior probabilities is calculated using Bayes' theorem: P(H|R)=P(R|H)P(H)/P(R), where P(H) is the prior and P(R) is a constant, and P(R|H) is easily calculated using the base QV to estimate the chance that a base is correct. If P=P(ref|R)+P(Hom|R)+P(Het|R), then the likelihood of each prediction is P(H|R)/P, and the hypothesis with the highest likelihood is called.

**Frequentist Method**

When coverage is high, we can use the frequentist method, which effectively estimates P(ref|R). If the genome position does not have an alternative allele (i.e., it has no SNP), we do this by estimating the expected number of reads matched to an alternative allele. Using the QV of all reads, we estimate the error rate of the bases covering this position. This error rate times the coverage depth is the number of non-reference reads expected. From the set of reads, we know the “actual” number of reads matching the alternative allele. A Poisson distribution is used to estimate the likelihood of this happening by chance. Low-frequency variants can be detected by this method. The variants are reported in a VCF file. SNPs called near homopolymer (HP) positions tend to be more likely to be false positives because of a higher chance of mismapping, in addition to the higher error rate at these positions. Also, longer HPs are more likely to cause false positives. We model this observation by adding an HP penalty. Formally, for any HP of length L we define a penalty function P(L)=a+bL when L<C and P(L)=a+bL+d otherwise, where a, b, c, d are user-specified parameters. In the frequentist method, the error rate of each base was determined by its base QV, and for any base belong to a HP of length L, we multiply P(L) by the error rate calculated from the base QV. The process is also equivalent to lowering the base QV of HP by a certain amount determined by length.
